# Unravelling the Mechanism of Rechargeable Aqueous Zn–MnO_2_ Batteries: Implementation of Charging Process by Electrodeposition of MnO_2_

**DOI:** 10.1002/cssc.202001216

**Published:** 2020-06-29

**Authors:** Jie Yang, Jianyun Cao, Yudong Peng, Wenji Yang, Suelen Barg, Zhu Liu, Ian A. Kinloch, Mark A. Bissett, Robert A. W. Dryfe

**Affiliations:** ^1^ Department of Chemistry University of Manchester Manchester M13 9PL UK; ^2^ National Graphene Institute University of Manchester Manchester M13 9PL UK; ^3^ Department of Materials University of Manchester Manchester M13 9PL UK

**Keywords:** aqueous Zn–MnO_2_ batteries, conversion, degradation, electrodeposition, Mn^2+^ dissolution

## Abstract

Poor cycling stability and mechanistic controversies have hindered the wider application of rechargeable aqueous Zn–MnO_2_ batteries. Herein, direct evidence was provided of the importance of Mn^2+^ in this type of battery by using a bespoke cell. Without pre‐addition of Mn^2+^, the cell exhibited an abnormal discharge–charge profile, meaning it functioned as a primary battery. By adjusting the Mn^2+^ content in the electrolyte, the cell recovered its charging ability through electrodeposition of MnO_2_. Additionally, a dynamic pH variation was observed during the discharge–charge process, with a precipitation of Zn_4_(OH)_6_(SO_4_)⋅5H_2_O buffering the pH of the electrolyte. Contrary to the conventional Zn^2+^ intercalation mechanism, MnO_2_ was first converted into MnOOH, which reverted to MnO_2_ through disproportionation, resulting in the dissolution of Mn^2+^. The charging process occurred by the electrodeposition of MnO_2_, thus improving the reversibility through the availability of Mn^2+^ ions in the solution.

## Graphical abstract

**Taking charge**: This work provides direct evidence of the importance of Mn^2+^ in rechargeable aqueous Zn–MnO_2_ batteries. The charging process is implemented by the electrodeposition of MnO_2_, thus improving the reversibility by the availability of Mn^2+^ in the solution. A conversion reaction between MnO_2_ and MnOOH occurs, rather than Zn^2+^ intercalation into MnO_2_.

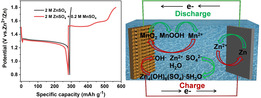

## Introduction

Electrochemical energy‐storage devices, that is, batteries, supercapacitors and hybrid devices (“supercapatteries”), play a crucial role in exploiting the electricity generated from renewable but intermittent energy sources such as wind and solar energy.^1^ Li‐ion batteries have achieved great commercial success in the rechargeable battery market because of their high energy density and good cycling stability.^2^ However, their cost along with concerns about safety and environmental impact significantly hinder their wider penetration to yield large‐scale applications,^3^ hence the drive to develop alternative battery chemistries such as Na‐ion batteries,^4^ K‐ion batteries,^5^ Mg‐ion batteries^6^ and Al‐ion batteries.^7^ Most of these alternative batteries still use flammable and toxic electrolytes, which motivates the study of aqueous batteries, in principle combining low cost, high safety and environmental friendliness. The alkaline primary Zn–MnO_2_ batteries have been commercialised and widely used for many years.^8^ However, this type of battery suffers from poor reversibility in alkaline electrolyte owing to the irreversible formation of byproducts on the cathode [i.e., Mn(OH)_2_, Mn_2_O_3_ and Mn_3_O_4_] and the anode [i.e., Zn(OH)_2_ and ZnO].^9^ Therefore, mild aqueous electrolytes used in the Zn–MnO_2_ battery are expected to enhance the electrochemical performance.

Recently, rechargeable aqueous Zn–MnO_2_ batteries have attracted attention: the above attributes of aqueous batteries add to their high theoretical specific capacity of 308 mAh g^−1^.^10^ To date, there have been notable developments in achieving high‐performance Zn–MnO_2_ batteries in mild aqueous electrolytes. However, the mechanism of the Zn–MnO_2_ battery is far from being fully understood and remains highly controversial. Various reaction mechanisms in Zn–MnO_2_ batteries have been proposed. The earliest reports of rechargeable Zn–MnO_2_ batteries described a mechanism based on reversible Zn^2+^ insertion/extraction into/from the tunnels of α‐MnO_2_.9e, 10a Subsequently, Liu and co‐workers proposed an alternative mechanism based on conversion between α‐MnO_2_ and H^+^ without zinc ion intercalation and de‐intercalation.^11^ Wang and co‐workers proposed that both processes operated, that is, the MnO_2_ cathode underwent successive H^+^ and Zn^2+^ insertion/extraction.^12^ Also, a precipitation of zinc hydroxide sulfate on the surface of α‐MnO_2_ was reported without zinc intercalation into the tunnels of MnO_2_.^13^ Recently, reversible chloride storage in Zn‐ion‐trapped Mn_3_O_4_ has been proposed by Jiang and Ji.^14^ In‐depth understanding of the mechanism in Zn–MnO_2_ batteries is important in optimising this battery chemistry in terms of performance and lifetime and thus promoting the large‐scale application of this type of battery.

As well as the differences in mechanism, various crystallographic polymorphs of manganese dioxide exist, such as α‐MnO_2_ (2×2), β‐MnO_2_ (1×1), δ‐MnO_2_ (1×∞) and todorokite‐MnO_2_ (3×3), which may also be responsible for the variety in reported charge‐storage mechanisms when the material is employed as an electrode.^11^, ^12^, ^13^, ^15^ Among them, α‐MnO_2_ with 2×2 tunnels has been of particular interest for Mn‐based Zn‐ion batteries. In common with other batteries, the performance of Zn–MnO_2_ batteries is generally investigated in coin cells with a small amount of electrolyte (≈100 μL), which complicates in operando measurements of mechanistically relevant parameters such as local variations in electrolyte pH and composition. Consequently, in this work, a custom cell was designed and used to reveal the reaction mechanism of the rechargeable aqueous Zn–MnO_2_ batteries, which enabled monitoring of variations in electrode potential and pH value of the electrolyte. The effect of solution‐phase Mn^2+^ on cell reversibility was explored: it was found that this species significantly changes the ability to charge the cell. The potential of the electrodes and pH of the electrolyte was monitored in situ during the discharge–charge process. The morphological and structural evolution of the α‐MnO_2_ cathode was also investigated during discharge–charge using SEM, XRD, TEM, scanning TEM energy‐dispersive spectroscopy (STEM‐EDS) and Raman spectroscopy. This combined structural and electrochemical study sheds light on the reaction mechanism of rechargeable aqueous Zn–MnO_2_ batteries.

## Results and Discussion

**Figure 1:**
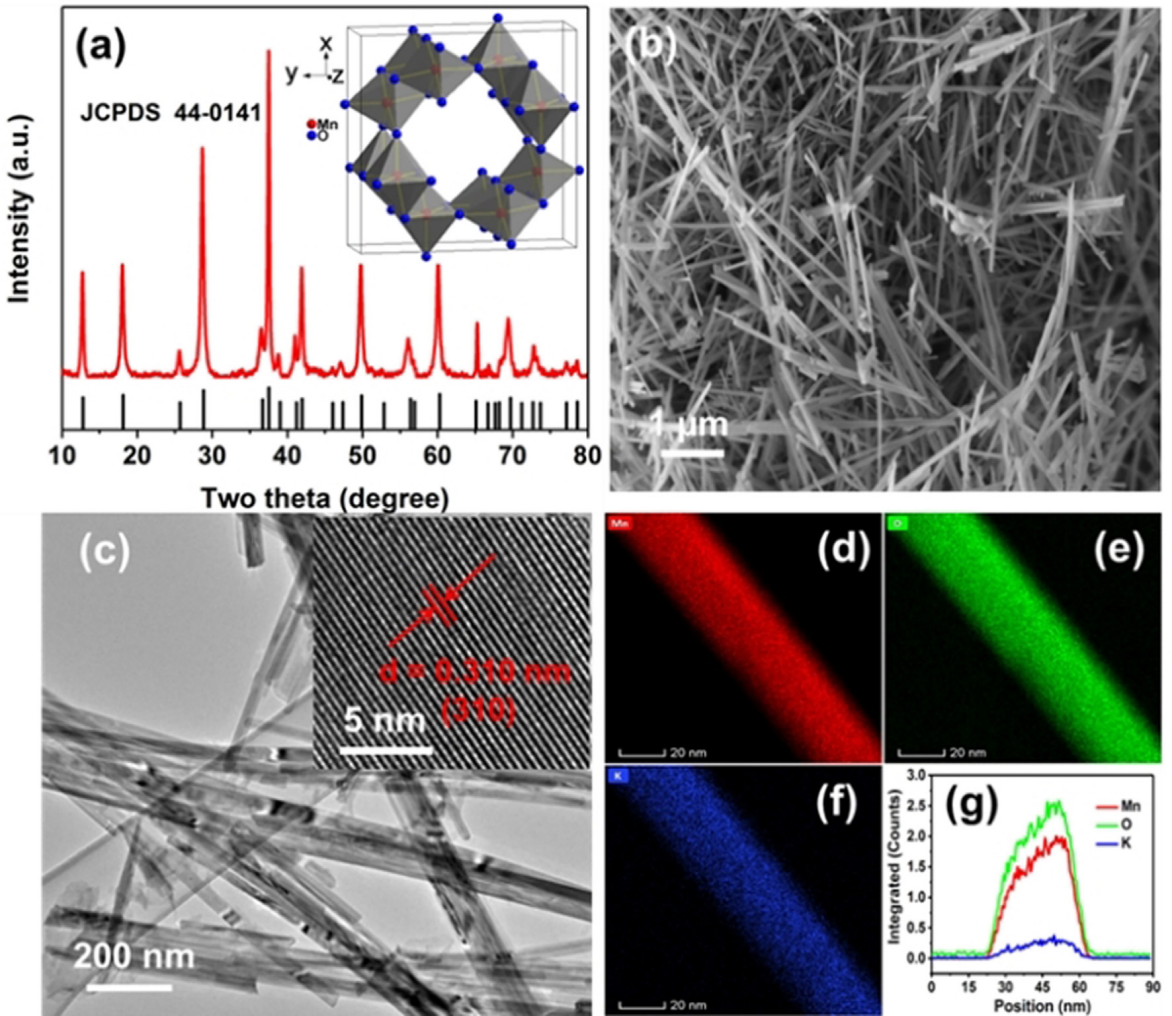
Characterizations of α‐MnO_2_. a) XRD pattern. b) Representative SEM image. c) Representative TEM image [inset showing the HRTEM image with a lattice distance of 0.310 nm corresponding to the (3 1 0) plane]. d–f) STEM‐EDS mappings of the elemental distributions of Mn, O and K in the MnO_2_. g) Line profiles of Mn, O and K across the MnO_2_ nanorod.

Figure 1 a shows the XRD pattern of the as‐prepared sample used as the cathode for Zn–MnO_2_ batteries, which is well indexed into the crystalline phase of α‐MnO_2_ (JCPDS: 44‐0141). The morphology was first observed by SEM in Figure 1 b, showing a homogeneous nanorod structure, with length ranging between 1 and 5 μm. The TEM further shows the detailed structure of nanorods with a diameter of 40±8 nm in Figure 1 c. The high‐resolution (HR)TEM image shows the α‐MnO_2_ nanorod with a well‐defined lattice constant of 0.310 nm for the (3 1 0) crystal plane, indicating its high degree of crystallinity. As shown in Figure 1 d–f, the corresponding STEM‐EDS mapping reveals abundant Mn, O and a low K content in the nanorod. The residual K is introduced into the MnO_2_ tunnels by the synthetic conditions owing to the KMnO_4_ starting material. Figure 1 g shows a profile of elements across the nanorod by linear scanning. The intensity profiles of the three elements (Mn, O and K) indicate a homogeneous distribution.

**Figure 2:**
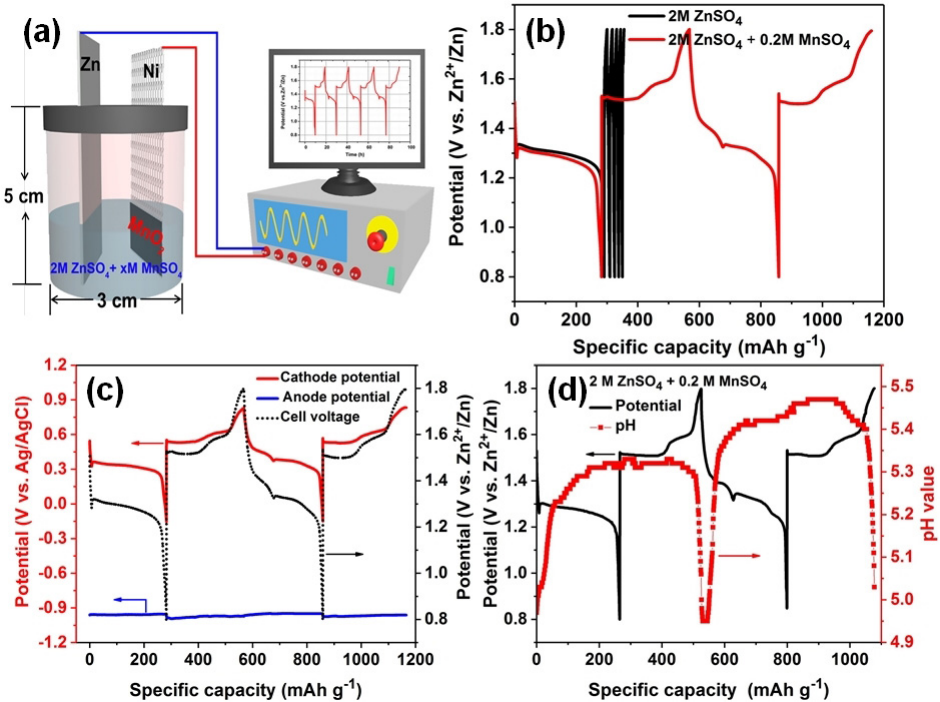
Tests in a home‐made cell composed of a working electrode (α‐MnO_2_), counter electrode (Zn) and electrolyte without separator. a) Schematic diagram of the cell. b) Galvanostatic discharge and charge curves in 2 m ZnSO_4_ and 2 m ZnSO_4_+0.2 m MnSO_4_. c) In situ potential monitoring. d) In situ pH monitoring.

Figure 2 a illustrates how the reaction mechanism was investigated by using a home‐made cell composed of α‐MnO_2_ as the cathode, Zn foil as the anode and 2 m ZnSO_4_ with different concentrations of MnSO_4_ as the electrolyte without separator. Although the cycling stability of the MnO_2_ electrode has been improved by adding Mn^2+^ to the electrolyte, the practical function of Mn^2+^ is far from well understood.10b, 11 To eliminate the effect of MnSO_4_, the pure 2 m ZnSO_4_ solution was first chosen as the electrolyte. Interestingly, we found that the cell was able to discharge with a high specific capacity of approximately 283 mAh g^−1^ and almost lost the ability to charge, shown in Figure 2 b. The subsequent cycles show a quite low reversible capacity of approximately 9 mAh g^−1^ (discharging for 0.3 h at 30 mA g^−1^) without the addition of MnSO_4_. Also, a brown deposit was observed on the current collector after charging, indicating the formation of MnO_2_, shown in Figures S1–S3 in the Supporting Information. Because there is no Mn^2+^ in the electrolyte, it is inferred that Mn^2+^ dissolves into the solution during the discharge process. The dissolved Mn^2+^ in the first discharge process was measured by inductively coupled plasma optical emission spectroscopy (ICP‐OES). The amount of Mn^2+^ in the electrolyte was calculated to be 0.774 mg, which is less than half of the total mass of Mn in the MnO_2_ cathode (2.8 mg), shown in Figure S4 in the Supporting Information. Also, previous literature has reported that the dissolution of Mn^2+^ results in a rapid capacity fade.^11^ Consequently, various concentrations of MnSO_4_ were added to the 2 m ZnSO_4_ electrolyte, as shown in Figure S5 in the Supporting Information. Significantly, there is no difference in the first discharge curve, but the charging curve begins to recover to a normal state compared with that using 2 m ZnSO_4_ electrolyte and the cell can then be cycled normally, as shown in Figure 2 b. This direct comparative experiment reveals that Mn^2+^ plays a vital role in the charging behaviour of the cell, not simply enhancing the cycling ability of the MnO_2_ electrode. This abnormal phenomenon requires us to reconsider the discharge and charge processes. If the discharge product is MnOOH or ZnMn_2_O_4_ as previously reported in the literature, then the cell should be charged normally to extract the inserted ions without addition of MnSO_4_, leading to a relatively high reversible capacity, contrary to what is observed in our study.

It is well‐known that manganese dioxide can be electrodeposited from Mn^2+^ aqueous solution by the following generalized reaction [Eq. (1)]:^16^

(1) $$\mathrm{Mn}{}^{2+}+2\;H{}_{2}O\to \mathrm{MnO}{}_{2}+4\;H{}^{+}+2\;e{}^{-}\;\;\;\left(E{}^{0}=1.23\;V\right)$$

Based on the Nernst equation, the theoretical potential to form MnO_2_ by electrodeposition in 0.2 m MnSO_4_ solution is calculated to be 1.413 V (vs. Zn^2+^/Zn), as shown in the Supporting Information. An in situ potential monitoring of each electrode was performed by using an Ag/AgCl reference electrode shown in Figure 2 c. The potential on the zinc anode is fairly consistent during the discharge–charge process. The potential on the cathode changes gradually during the discharge process, whereas it dramatically jumps to 1.520 V (vs. Zn^2+^/Zn) at the beginning of the charge process. This potential is consistent with the required theoretical potential of MnO_2_ electrodeposition, suggesting a possible electrodeposition of MnO_2_. Also, the electrodeposition of MnO_2_ would cause pH variations in the solution; therefore, an in situ pH monitoring of the electrolyte during the discharge–charge process was also performed. As shown in Figure 2 d, the electrolyte pH value increases as discharge proceeds. It reaches a pH of 5.33 at the end of discharge with a plateau region around this value. Upon charging, there is a slow decrease in the pH value, and it goes back to the initial value at the end of the charge process. During the charge process, the variation in pH provides evidence that the charging process is probably based on the electrodeposition of MnO_2_. To give further support for this, the electrodeposition of MnO_2_ was investigated with a bare nickel mesh as working electrode, a Zn foil as counter electrode and 2 m ZnSO_4_+0.2 m MnSO_4_ as electrolyte.

As shown in Figure S6a in the Supporting Information, the charge–discharge profile for the bare nickel mesh is quite similar to that in the Zn–MnO_2_ battery although there is no active material loaded on the cathode. Without MnSO_4_ additive, the bare nickel mesh only exhibits a capacitor‐like behaviour (Figure S6 b in the Supporting Information), indicating that the MnO_2_ electrodeposited from the solution acts as the active material. Considering that the cell cannot normally charge without the Mn^2+^ as additive and the onset charging potential is quite close to the electrodeposition potential of MnO_2_ coupled with the trend in pH value during the charging process, it is speculated that charging is based on the electrodeposition of MnO_2_ on the cathode. The pre‐addition of MnSO_4_ in the solution can provide enough Mn for electrodeposition of MnO_2_ to compensate for the loss of MnO_2_ from the electrode.

**Figure 3:**
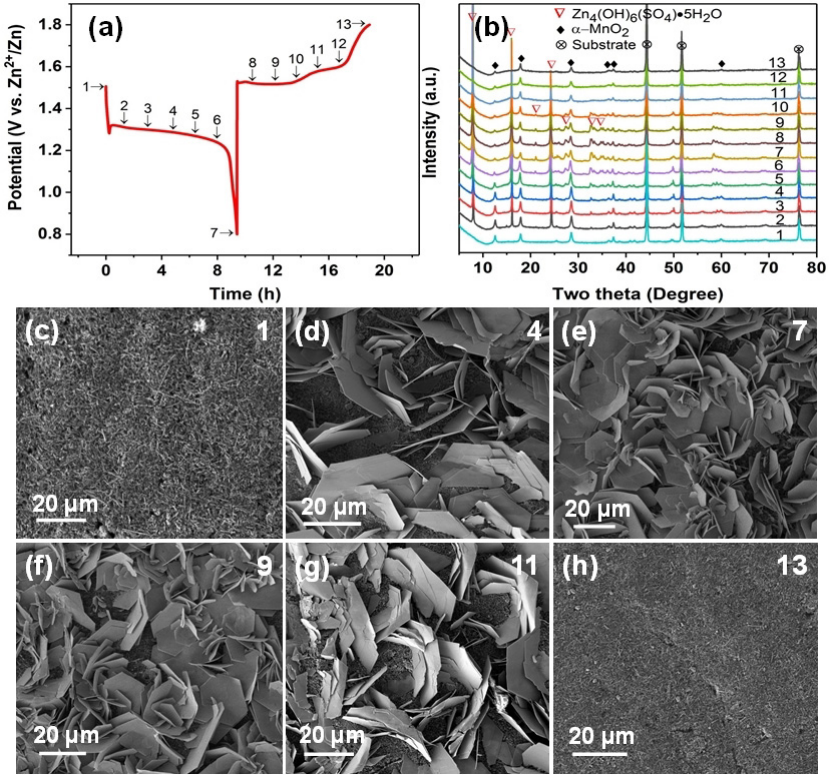
a) Typical profile during the first discharge–charge process at 30 mA g^−1^. b) Evolution of ex situ XRD patterns of MnO_2_ electrodes recorded at different states denoted in a). c–h) Corresponding SEM images of MnO_2_ electrodes collected at states denoted by 1, 4, 7, 9, 11 and 13.

Ex situ XRD and SEM data was acquired for the α‐MnO_2_ cathode in the 2 m ZnSO_4_+0.2 MnSO_4_ electrolyte to reveal the phase and morphology evolution, respectively. As shown in Figure 3 a, b, some emerging peaks at approximately 8.0, 16.0, 21.1, 24.4, 27.3, 32.7 and 34.7° are well matched to Zn_4_(OH)_6_(SO_4_)⋅5 H_2_O (JCPDS: 78‐0246) during the discharging process. The zinc hydroxide sulfate hydrate [Zn_4_(OH)_6_(SO_4_)⋅*n* H_2_O, *n*=0, 0.5, 1, 3, 4 and 5, ZHSH] consists of stacked zinc hydroxide layers. The interlayer space is filled with zinc sulfate and different numbers of water molecules, resulting in an interlayer distance of 7–11 Å.^13^, ^17^ When the cell is further discharged to 0.8 V, the corresponding peak intensity of Zn_4_(OH)_6_(SO_4_)⋅5 H_2_O shows an upward trend, indicating its growth. During the subsequent charging process, the intensity of Zn_4_(OH)_6_(SO_4_)⋅5 H_2_O peaks gradually decreases, and finally the corresponding peaks disappear, suggesting a reversible precipitation/dissolution of Zn_4_(OH)_6_(SO_4_)⋅5 H_2_O. As shown in Figure 3 c, the original MnO_2_ electrode exhibits a clean surface. After discharging, some large flakes emerge on the electrode surface (Figure 3 d, e). EDS analysis shows that the flake‐like product contains abundant Zn, O and S (Figure S7 in the Supporting Information). During the subsequent charging process, the large flakes gradually disappear (Figure 3 f–h). The reversible morphological evolution during the discharge–charge process is consistent with the XRD results. In fact, the formation of this material has been independently reported by other groups.^11^, ^13^, ^18^ It is worth noting that the formation of zinc hydroxide sulfate has been rationalised in various ways. Liu and co‐workers^11^ think that with the consumption of H^+^ in the electrolyte, the increasing concentration of OH^−^ leads to the formation of zinc hydroxide sulfate hydrate. On this basis they proposed a new mechanism based on the conversion reaction between MnO_2_ and H^+^ without zinc ion intercalation and de‐intercalation. Oh and co‐workers^13^ attribute the precipitation of zinc hydroxide sulfate hydrate to the disproportionation of the unstable trivalent manganese. The dissolution of Mn^2+^ into the solution leads to an increase in the pH of the solution, thus triggering precipitation of zinc hydroxide sulfate hydrate on the electrode surface. There is no doubt that the formation of zinc hydroxide sulfate hydrate is intimately bound to variation in solution pH, but the reason for the pH variation is unclear. Also, the point at which the zinc hydroxide sulfate forms has not been mentioned. According to ex situ XRD results from Xia and co‐workers,^18^ the zinc hydroxide sulfate hydrate flakes do not form in the potential region from 1.8 to 1.35 V (vs. Zn^2+^/Zn) and start to emerge with further discharging to 1 V (vs. Zn^2+^/Zn). During the subsequent charging, the zinc hydroxide sulfate hydrate gradually vanishes. To confirm that the formation of zinc hydroxide sulfate hydrate is pH‐dependent, the discharged electrode was washed with acetic acid. As shown in Figure S8 in the Supporting Information, it is found that the flakes on the electrode surface can be totally removed, verifying the pH‐dependent property of zinc hydroxide sulfate hydrate. It is also observed that there are far fewer MnO_2_ nanorods on the surface, indicating the consumption of MnO_2_ during the discharge process. Also, the XRD pattern of the discharged product was compared with the pure ZnMn_2_O_4_, which does not demonstrate a good match with reported intercalated phases in the literature, shown in Figure S9 a, b in the Supporting Information. Further, the XRD pattern of the discharged product was compared with pure MnOOH, shown in Figure S9 c, d in the Supporting Information. It was previously reported that the MnOOH phase as a discharged product was submerged in the strong characteristic peaks of current collector (carbon paper) and resultant products.^11^ However, the strongest peak of MnOOH at 26.3° was not detected in our study. The peaks from zinc hydroxide sulfate hydrate dominate the XRD patterns of the discharged product, and neither MnOOH nor ZnMn_2_O_4_ can be detected. Because the electrode surface is covered by the zinc hydroxide sulfate hydrate, the complex peaks complicate the determination of the structural evolution of MnO_2_. Therefore, the fully discharged electrode was washed with acetic acid to remove the precipitated zinc hydroxide sulfate hydrate. Surprisingly, only α‐MnO_2_ can be observed, and no evidence for Zn^2+^ or proton intercalation into α‐MnO_2_ can be found, as shown in Figure S10 in the Supporting Information. This is consistent with the report by Oh and coworkers.^13^ Consequently, it is more likely that the Zn ions cannot intercalate into the tunnels of α‐MnO_2_ to form intercalated phases; this point was further investigated by TEM and Raman spectroscopy (see below). The phase and morphology evolution of α‐MnO_2_ with pure 2 m ZnSO_4_ electrolyte was also investigated, shown in Figure S11 in the Supporting Information. With regard to MnOOH, it is probably unstable owing to the Jahn–Teller effect of the Mn^3+^ ion, thus forming solid MnO_2_ and aqueous Mn^2+^.^13^, ^19^ This shows a good match with the observation that there is a brown deposit on the current collector during the charge process although Mn^2+^ is not pre‐added to the solution. Additionally, this is consistent with the XRD result after the zinc hydroxide sulfate hydrate flakes are removed with the weak acid. A previous report suggested that there is a phase transformation between α‐MnO_2_ and Zn‐buserite.15c However, it should be noted that XRD patterns of zinc hydroxide sulfate hydrate phase are quite similar to the previously reported Zn‐birnessite.^13^, 15d Therefore, more attention should be paid to analyse the XRD results, and other techniques are required to further elucidate the reaction mechanism of α‐MnO_2_.

**Figure 4:**
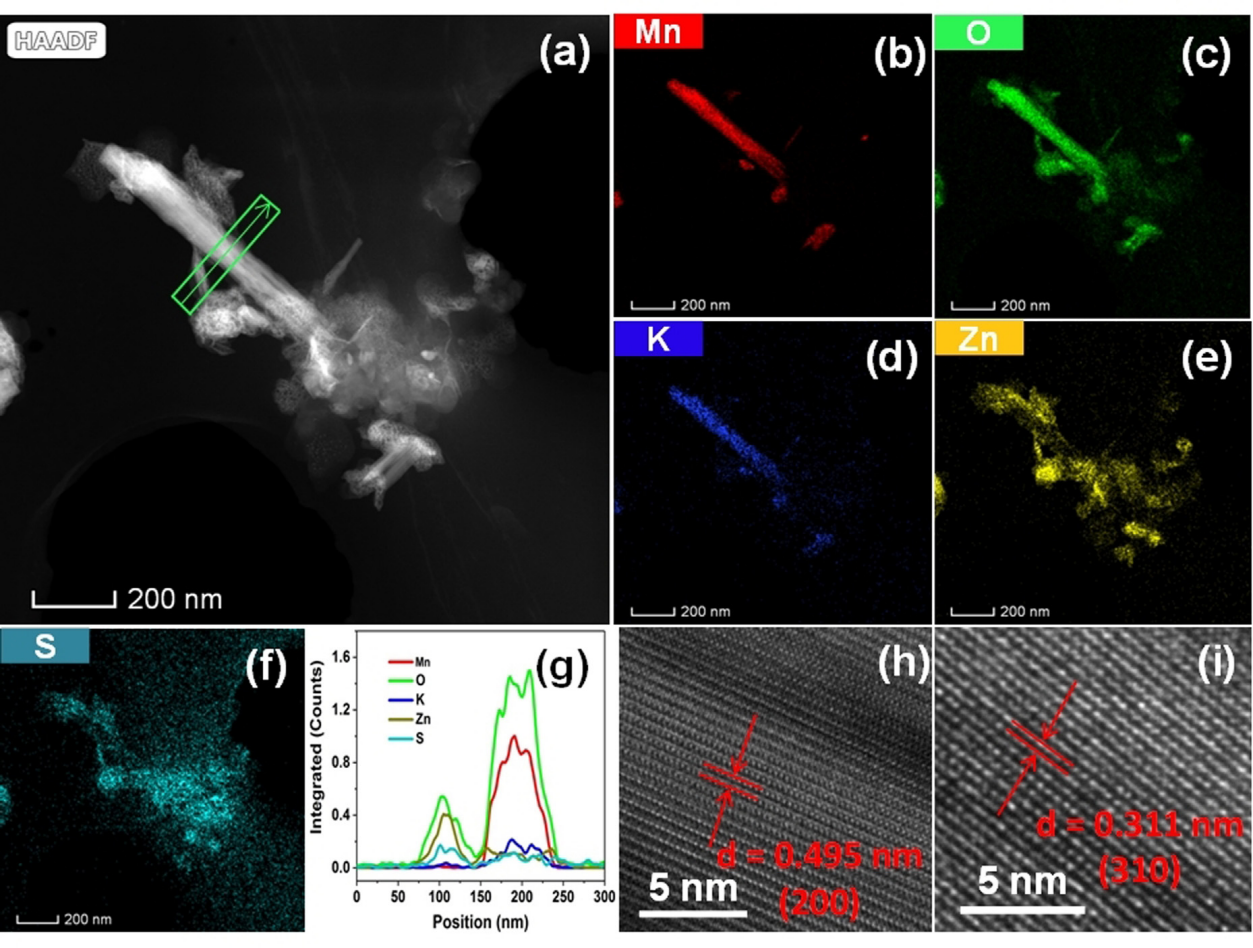
TEM/HRTEM images of MnO_2_ electrodes after first discharge. a) STEM‐HAADF (high‐angle annular dark‐field) image of short nanorods. b–f) STEM‐EDS mappings of different elements. g) Line profiles of different elements across the α‐MnO_2_ nanorod. h, i) HRTEM images.

TEM was used to gain further insight into the structural evolution of α‐MnO_2_ during discharge process. As shown in Figure 4 a (also in Figure S12 in the Supporting Information), the morphology of the α‐MnO_2_ electrode is well maintained when it is first discharged to 0.8 V. The corresponding STEM‐EDS mapping reveals various elements such as Mn, O, K, Zn and S in the discharge products, shown in Figure 4 b–f. The Mn, O and K elements simultaneously coexist in the same region whereas Zn, O and S elements simultaneously coexist in a different region. Previous papers reported that zinc ions intercalate into the tunnel of MnO_2_ to form the spinel ZnMn_2_O_4_ or a tunnel or layered Zn_*x*_MnO_2_ phase.9e, 10a, 15c However, it is found that Zn is not homogeneously distributed in the bulk of the MnO_2_ and only exists on the surface of the MnO_2_ nanorod. The Zn element shows a similar distribution to that of S, indicating the formation of zinc hydroxide sulfate, which is consistent with results of SEM and XRD. To further confirm the localized elemental distribution, the corresponding linear scanning position is shown in the highlighted rectangle. As shown in Figure 4 g, Mn, O and K clearly exhibit a synchronous trend whereas Zn, S show a different synchronous trend, indicating that the zinc ions do not intercalate into the tunnel of MnO_2_. HRTEM was further used to reveal the lattice distance of the discharged products, shown in Figure 4 h, i. A larger lattice distance of 0.495 nm is indexed to the (2 0 0) plane of α‐MnO_2_ and a smaller lattice distance of 0.311 nm is consistent with the (3 1 0) plane of α‐MnO_2_. The TEM results showed that the lattice fringes from the discharged products do not match the reported intercalated phases in the literature. Also, the synthesized pure ZnMn_2_O_4_ (Figure S13 in the Supporting Information) was investigated by TEM. The corresponding STEM‐EDS mapping shows a homogeneous distribution of Zn, Mn and O, shown in Figures S14 and S15. The lattice distance was revealed by HRTEM, showing a 0.485 nm corresponding to the (1 0 1) plane of ZnMn_2_O_4_, shown in Figure S16 in the Supporting Information. Furthermore, the electrochemical performance of the ZnMn_2_O_4_ was investigated by galvanostatic charge–discharge method. It is found that the ZnMn_2_O_4_ does not exhibit electrochemical activity with an extremely low specific capacity of 15 mAh g^−1^, as shown in Figure S17 in the Supporting Information. The charge–discharge profile, showing a capacitor‐like behaviour, suggests that the zinc ions in the spinel ZnMn_2_O_4_ cannot be extracted from the tunnel, which provides an indirect proof to support our conclusion that the mechanism does not involve the insertion of zinc ions. Steingart and co‐workers reported that ZnMn_2_O_4_ is electrochemically inert, which is consistent with our results.^20^ Although Zn^2+^ has a relatively small ionic radius of 0.75 Å, it is still difficult to find ideal cathode materials to accommodate Zn^2+^ owing to the strong electrostatic interaction between divalent Zn^2+^ and solid framework of host materials as well as the difficulty in redistributing the multiple charges of the inserted cations in the host.^13^, ^21^ Additionally, the high ionic radius of hydrated Zn^2+^ (Table S1 in the Supporting Information) will add an additional barrier for the intercalation process despite the partial charge shielding of the solvation shell.15d Therefore, our conclusion is that it is difficult for zinc ions to intercalate into the tunnel of α‐MnO_2_.

Although the test capacity may be affected by various factors such as the properties (defects, surface area) of MnO_2_, the composition of the electrode and measurement conditions such as discharge current and electrolyte, the capacity obtained at a low current density is more likely to approach the theoretical capacity of the electrode materials. The practical specific capacity of MnO_2_ reported by most researchers is less than the theoretical capacity of 308 mAh g^−1^,10c, 22 which is consistent with our results. Although the MnO_2_/a‐CNT (a‐CNT: acid‐treated carbon nanotubes) nanocomposites displayed an ultrahigh capacity of 665 mAh g^−1^ at 0.1 Ag^−1^, the improved capacity was attributed to the numerous oxidative functional groups in the a‐CNT, which could act as additional storage sites.^23^ Based on the statistical principle and experimental errors, the Faradaic reaction is more likely to be based on a one‐electron transfer reaction (Mn^4+^+e^−^→Mn^3+^) in this battery system. It means that the MnO_2_ will not directly transform into Mn^2+^ although we have directly observed the dissolution of Mn^2+^ into the solution. The intermediate is likely to be MnOOH. Previous observations showed that MnOOH dissolved significantly below pH 6 through an acidic disproportionation owing to the high‐spin electronic configuration.19a, 24 It will form dissolved Mn^2+^ and solid MnO_2_ [2 Mn^3+^→Mn^2+^(aq)+Mn^4+^(s)] owing to the Jahn–Teller effect.^13^, 19b During the whole discharge process, the pH of the solution is always below 6, which will facilitate the transformation of MnOOH into Mn^2+^ and MnO_2_. This is probably the reason for the difficulty in detecting the intermediate MnOOH. It should be noted that this disproportionation involves a localized electron transfer without contributing any capacity.

Based on the above analysis, the reaction mechanism of this battery during the discharge process including the intermediate Mn^3+^ can described as follows:

Reductive half‐reaction [Eqs. (2)–(2)]:

(2) $$\mathrm{MnO}{}_{2}\left(s\right)+H{}^{+}\left(\mathrm{aq}\right)+e{}^{-}\to \mathrm{MnOOH}\left(s\right)$$

(3) $$2\;\mathrm{MnOOH}\left(s\right)+2\;H{}^{+}\left(\mathrm{aq}\right)\to \mathrm{MnO}{}_{2}\left(s\right)+\mathrm{Mn}{}^{2+}\left(\mathrm{aq}\right)+2\;H{}_{2}O\left(l\right)$$

(4) $$4\;\mathrm{Zn}{}^{2+}\left(\mathrm{aq}\right)+6\;\mathrm{OH}{}^{-}\left(\mathrm{aq}\right)+\mathrm{SO}{}_{4}{}^{2-}\left(\mathrm{aq}\right)+5\;H{}_{2}O\left(l\right)\to \mathrm{Zn}{}_{4}\left(\mathrm{OH}\right){}_{6}\left(\mathrm{SO}{}_{4}\right)\cdot 5\;H{}_{2}O\left(s\right)↓$$

Oxidative half‐reaction [Eq. (5)]:

(5) $$\mathrm{Zn}\left(s\right)\to \mathrm{Zn}{}^{2+}\left(\mathrm{aq}\right)+2\;e{}^{-}$$

Overall [Eq. (6)]:

(6) $$4\;\mathrm{MnO}{}_{2}\left(s\right)+10\;H{}^{+}\left(\mathrm{aq}\right)+4\;\mathrm{Zn}\left(s\right)+\mathrm{SO}{}_{4}{}^{2-}\left(\mathrm{aq}\right)+3\;H{}_{2}O\left(l\right)\to 4\;\mathrm{Mn}{}^{2+}\left(\mathrm{aq}\right)+\mathrm{Zn}{}_{4}\left(\mathrm{OH}\right){}_{6}\left(\mathrm{SO}{}_{4}\right)\cdot 5\;H{}_{2}O\left(s\right)↓$$

During the charge process, the reaction mechanism can be formulated as follows:

Oxidative half‐reaction [Eqs. (7) and (7)]:

(7) $$\mathrm{Mn}{}^{2+}\left(\mathrm{aq}\right)+2\;H{}_{2}O\left(l\right)\to \mathrm{MnO}{}_{2}\left(s\right)+4\;H{}^{+}\left(\mathrm{aq}\right)+2\;e{}^{-}$$

(8) $$\mathrm{Zn}{}_{4}\left(\mathrm{OH}\right){}_{6}\left(\mathrm{SO}{}_{4}\right)\cdot 5\;H{}_{2}O\left(s\right)+6\;H{}^{+}\left(\mathrm{aq}\right)\to 4\;\mathrm{Zn}{}^{2+}\left(\mathrm{aq}\right)+\mathrm{SO}{}_{4}{}^{2-}\left(\mathrm{aq}\right)+11\;H{}_{2}O\left(l\right)$$

Reductive half‐reaction [Eq. (9)]:

(9) $$\mathrm{Zn}{}^{2+}\left(\mathrm{aq}\right)+2\;e{}^{-}\to \mathrm{Zn}\left(s\right)$$

Overall [Eq. (10)]:

(10) $$4\;\mathrm{Mn}{}^{2+}\left(\mathrm{aq}\right)+\mathrm{Zn}{}_{4}\left(\mathrm{OH}\right){}_{6}\left(\mathrm{SO}{}_{4}\right)\cdot 5\;H{}_{2}O\left(s\right)\to 4\;\mathrm{MnO}{}_{2}\left(s\right)+10\;H{}^{+}\left(\mathrm{aq}\right)+4\;\mathrm{Zn}\left(s\right)+\mathrm{SO}{}_{4}{}^{2-}\left(\mathrm{aq}\right)+3\;H{}_{2}O\left(l\right)$$

Although this aqueous battery is safe and environmentally friendly compared with organic‐based batteries, the key conversion reaction is more complicated than that in intercalation chemistry because it is accompanied by MnOOH disproportionation and a precipitation reaction. The accessibility of Mn^2+^ in the solution is crucial to improving the reversibility of this battery, and the pH of the electrolyte needs to be controlled. The precipitation reaction plays a vital role in dynamically tuning the pH of the solution. If the pH is higher than approximately 7 (alkaline solution), this type of battery functions as an alkaline primary battery. If the pH is lower than approximately 3 (acid solution), the zinc metal is unstable with respect to the generation of hydrogen gas. This fundamental research is expected to provide useful guidance in promoting the practical application of rechargeable aqueous Zn–MnO_2_ batteries.

## Conclusions

We have provided direct evidence of the importance of Mn^2+^ in rechargeable aqueous Zn–MnO_2_ batteries by studying their discharge–charge behaviour. The reaction mechanism was studied with a home‐made cell containing a large amount of electrolyte (≈12 mL) without separator. In the absence of pre‐added Mn^2+^ in the electrolyte, the dissolved Mn^2+^ ions are not easily returned to the electrode, leading to the instantaneous failure of the cell. This provides a direct way to identify the degradation mechanism of the rechargeable aqueous Zn–MnO_2_ batteries, thus helping to reveal the underlying reaction mechanism. By increasing the content of Mn^2+^ in the electrolyte, the loss of MnO_2_ from the electrode can be replenished by electrodeposition of MnO_2_ from the pre‐addition Mn^2+^ in the electrolyte, leading to a normal charging behaviour. This degradation and charging mechanism also applies to other manganese oxides such as β‐MnO_2_. More interestingly, the in‐depth investigation of morphology and structure of the discharged cathode reveals that there is a conversion reaction between MnO_2_ and MnOOH without zinc ion intercalation into the tunnel of α‐MnO_2_ to form the ZnMn_2_O_4_ or Zn_*x*_MnO_2_ phase. The dissolution of Mn^2+^ is caused by the acidic disproportionation of MnOOH. The precipitation of zinc hydroxide sulfate hydrate is essential to buffer the pH value of the solution. These findings shed light on the degradation and reaction mechanism of rechargeable aqueous Zn–MnO_2_ batteries and provide useful guidance in designing high‐performance rechargeable aqueous Zn–MnO_2_ batteries.

## Conflict of interest

*The authors declare no conflict of interest*.

## Supporting information

As a service to our authors and readers, this journal provides supporting information supplied by the authors. Such materials are peer reviewed and may be re‐organized for online delivery, but are not copy‐edited or typeset. Technical support issues arising from supporting information (other than missing files) should be addressed to the authors.

Supplementary
