# Supplementary material for: Unravelling the Mechanism of Rechargeable Aqueous Zn–MnO2 Batteries: Implementation of Charging Process by Electrodeposition of MnO2
Source: ChemSusChem. 2020 Jun 29;13(16):4103–10. doi: 10.1002/cssc.202001216 (PMC7496518; doi:10.1002/cssc.202001216)
Supplement: Supplementary file 1 — Supplementary [file CSSC-13-4103-s001.pdf]

ChemSusChem

Supporting Information

Unravelling the Mechanism of Rechargeable Aqueous Zn–MnO_2 Batteries: Implementation of Charging Process by Electrodeposition of MnO_2

Jie Yang,[a, b] Jianyun Cao,[c] Yudong Peng,[c] Wenji Yang,[c] Suelen Barg,[c] Zhu Liu,[c]
Ian A. Kinloch,[c] Mark A. Bissett,*[c] and Robert A. W. Dryfe*[a, b]

Table of Contents

1. Experimental Procedures 2

1.1 Synthesis of MnO_2 and $ZnMn_2O_4$. 2

1.2 Characterization....................2

1.3 Electrochemical tests. 2

2. Results and Discussion 3

3. Potential conversion 20

4. References....................20

1. Experimental Procedures

1.1 Synthesis of MnO_2 and $ZnMn_2O_4$. α-MnO_2 nanorods were prepared by a typical hydrothermal method.[1] Briefly, 0.50 g of $KMnO_4$ and 0.21 g of $MnSO_4·H_2O$ were added to 32 mL deionized water to form a homogeneous solution under magnetic stirring for about 30 min. The solution was then transferred to a Teflon-lined stainless steel autoclave with a volume of 50 mL and heated in an oven at 160 °C for 12 h. The obtained product was collected by filtration, washed with deionized water, and dried at 80 °C in an oven overnight. The corresponding reaction can be formulated as follows: [2]

$$2KMnO_4 + 3MnSO_4 + 2H_2O \rightarrow 5MnO_2 + K_2SO_4 + 2H_2SO_4$$

The $ZnMn_2O_4$ was prepared by a self-propagating combustion method.[3] Typically, zinc nitrate hexahydrate (14.874 g) and manganese nitrate (17.895 g) were dissolved into 100 mL water containing citric acid (43.228 g) to form a homogeneous solution with stirring. Then the solution was heated to obtain a gel precursor which was further heat-treated using a hotplate, with a set temperature of 300 °C, until it combusted. After a self-propagating combustion, the precursor was further treated in a muffle furnace under 700 °C for 2h to obtain a fine powder.

1.2 Characterization. The crystal structure of the materials was characterized by Powder X-ray diffraction (XRD) using a Bruker D8 Advanced diffractometer with Cu-Kα radiation (λ = 1.5406 Å). The morphologies and structures were characterized by field-emission scanning electron microscopy (FESEM, TESCAN Mira3) and transmission electron microscopy (TEM, FEI Talos F200X). The Raman spectra were obtained by using a Renishaw inVia Raman instrument with an excitation wavelength of 532 nm. Elemental analysis of Mn in the electrolyte was conducted by Inductively Coupled Plasma Optical Emission Spectroscopy (ICP-OES) using a “PlasmaQuant” model PQ 9000.

1.3 Electrochemical tests. Electrodes were prepared by mixing 70 wt% of MnO_2 as active material with 20 wt% of carbon black (“Super-P”, conductive additive) and 10 wt% of binder polytetrafluoroethylene (PTFE). The MnO_2 powder and carbon black were firstly dispersed and mixed in isopropanol. Then PTFE was added to get a paste which was rolled into a uniform film with thickness about 50 µm and dried at 80 °C for 24 h. Afterwards, the film was cut into smaller pieces 0.8 cm × 0.8 cm in size and about 3-4 mg in weight. The areal loading density of MnO_2 is 5-6 mg cm^{-2}. Finally, the small films were pressed on a nickel mesh as working electrode (Nickel mesh, Advent Research Materials). The electrolyte was deoxygenated by using nitrogen gas for 1h before use. For the mechanistic investigation, a home-made cell is composed of a working electrode (MnO_2, 0.8 cm × 0.8 cm), counter electrode (zinc foil 1.5 cm × 1.5 cm with a mass loading of about 191 mg cm^{-2}) and electrolyte (2 M $ZnSO_4$ or 2 M $ZnSO_4$ + 0.2 M $MnSO_4$, about 12 mL) without separator. For the in-situ test, an Ag/AgCl reference electrode and a pH meter were placed between the working electrode and the counter electrode. During the discharge-charge process, the pH value of the electrolyte was recorded every minute using a digital pH meter. Galvanostatic charge/discharge performances were conducted on a battery test

system (Basytec, Germany). Cyclic voltammetry measurements were carried out using an AUTOLAB potentiostat. All the tests were performed at room temperature.

2. Results and Discussion

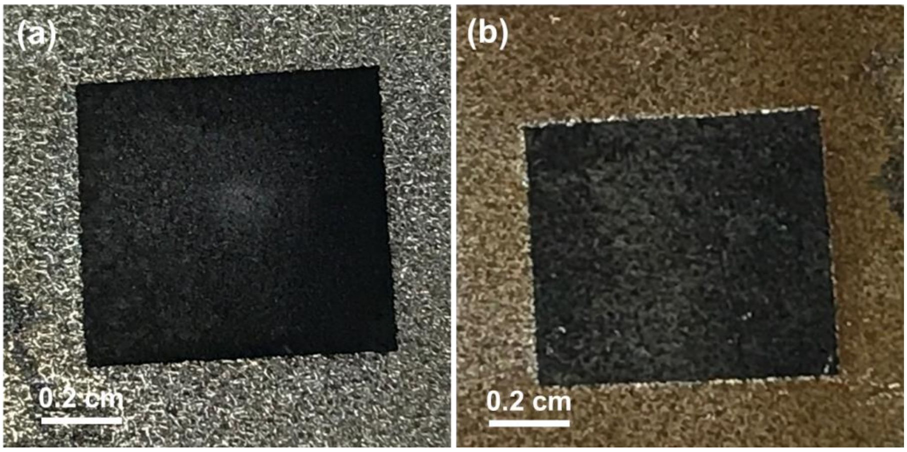

Figure S1. Digital images of the electrode: (a) after 1st discharge, (b) after 1st charge

After charging, the bare nickel mesh is covered by the brown deposit.

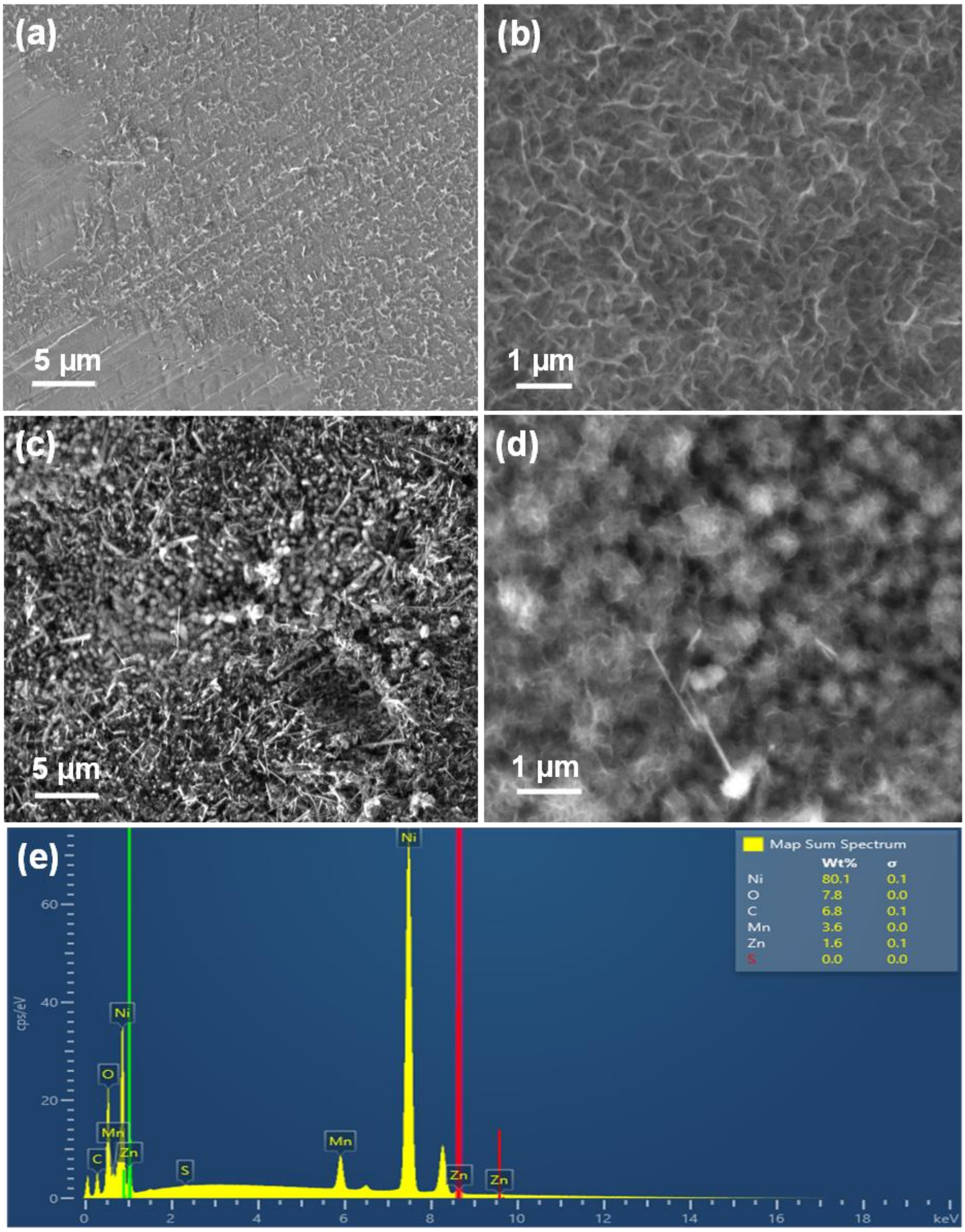

Figure S2. SEM images of the charged electrode: (a-b) the surface of bare current collector, (c-d) the surface of α-MnO_2 electrode, (e) energy-dispersive spectroscopy (EDS) analysis of brown deposit

The brown deposit was investigated by SEM, exhibiting a spongiform structure in Figure S2. The corresponding morphology is quite common in MnO_2.[4] Based on the colour and morphology as well as the elemental composition, it is inferred that this brown deposit is MnO_2. Furthermore, the brown deposit was investigated by Raman spectroscopy.

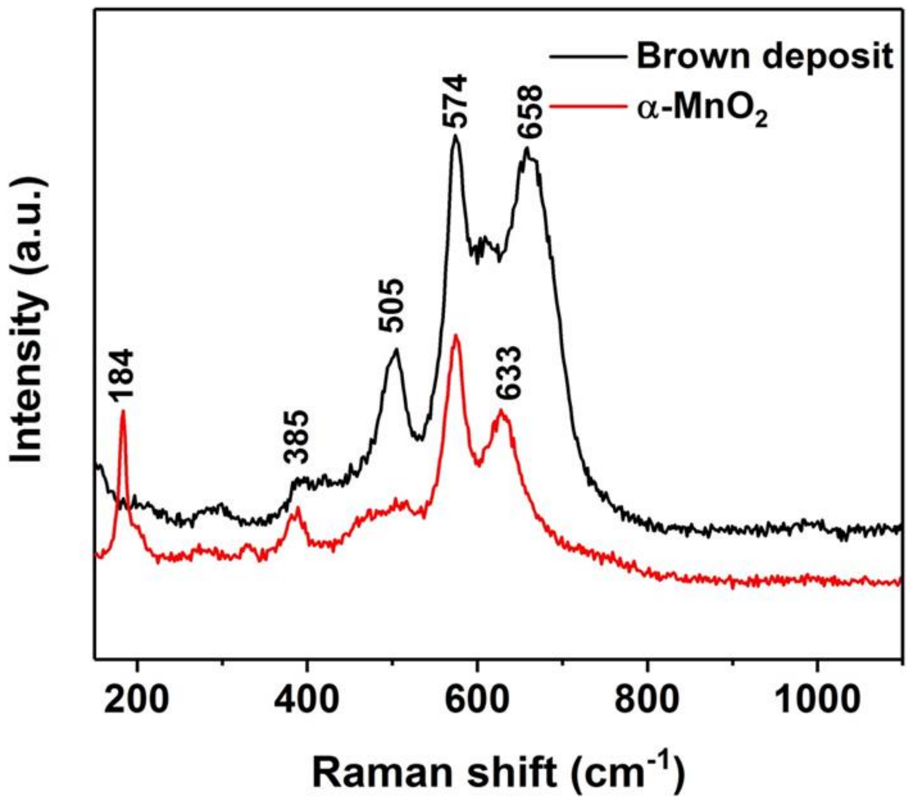

Figure S3. Raman spectra of brown deposit and α-MnO_2

According to the Raman spectrum, the brown deposit formed on the current collector can be ascribed to birnessite-type MnO_2.[5]

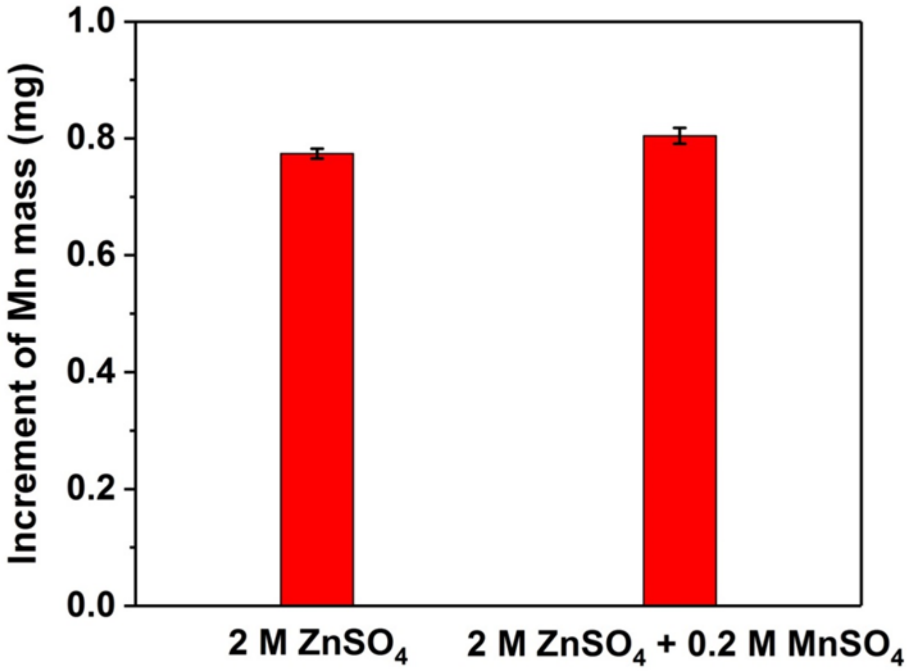

Figure S4. The increment of Mn^{2+} in the solution after 1st discharge

It has been reported that the pre-addition of Mn^{2+} to the electrolyte enables the improved cycling stability of MnO_2 electrode by inhibiting the dissolution of Mn^{2+}.[6] This is not the case in our experiments. Since the dissolution of Mn^{2+} is caused by disproportionation due to high-spin electronic configuration of Mn^{3+},[7] the pre-addition of Mn^{2+} in the solution will not affect the configuration of Mn^{3+}, thus is unable to suppress the dissolution of Mn^{2+}. The pre-addition of Mn^{2+} in the solution can provide enough Mn^{2+} for electrodeposition into MnO_2 to compensate the loss of MnO_2 from the electrode.

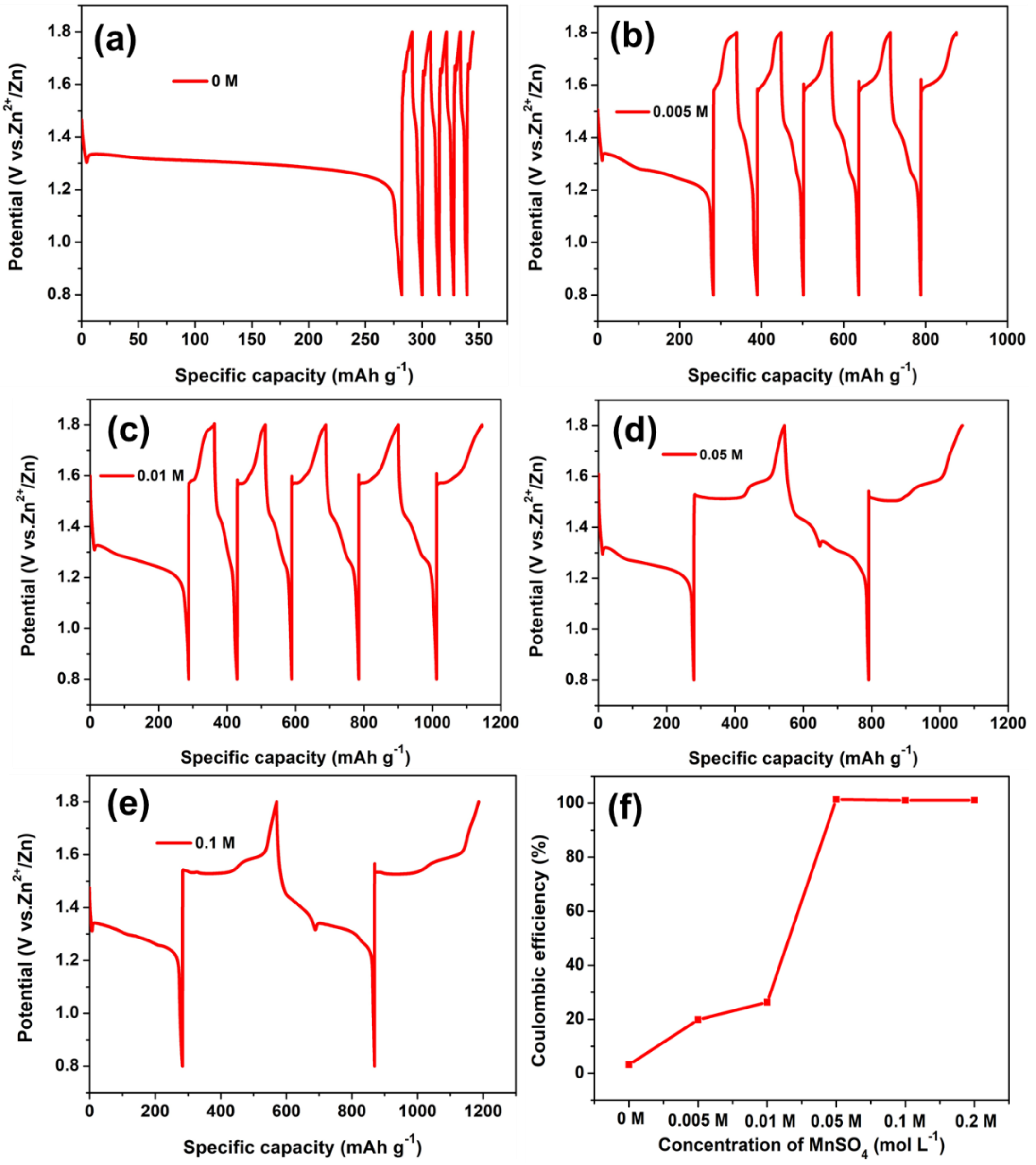

Figure S5. Typical galvanostatic charge-discharge curves of the cells with different concentrations of $MnSO_4$ in the electrolyte at 30 mA g^{-1}, (a) 0 M, (b) 0.005 M, (c) 0.01 M, (d) 0.05 M, (e) 0.1 M, (f) the corresponding coulombic efficiency in the first cycle as a function of concentration of $MnSO_4$ additive.

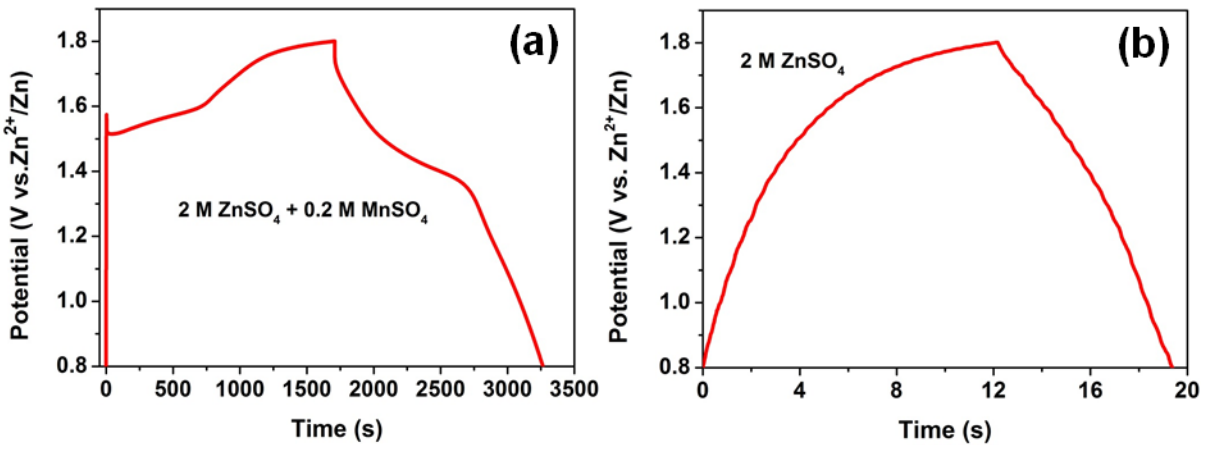

Figure S6. Typical galvanostatic charge-discharge curves of bare nickel mesh at current of 100 μA in (a) 2 M $ZnSO_4$ + 0.2 M $MnSO_4$ and (b) 2 M $ZnSO_4$

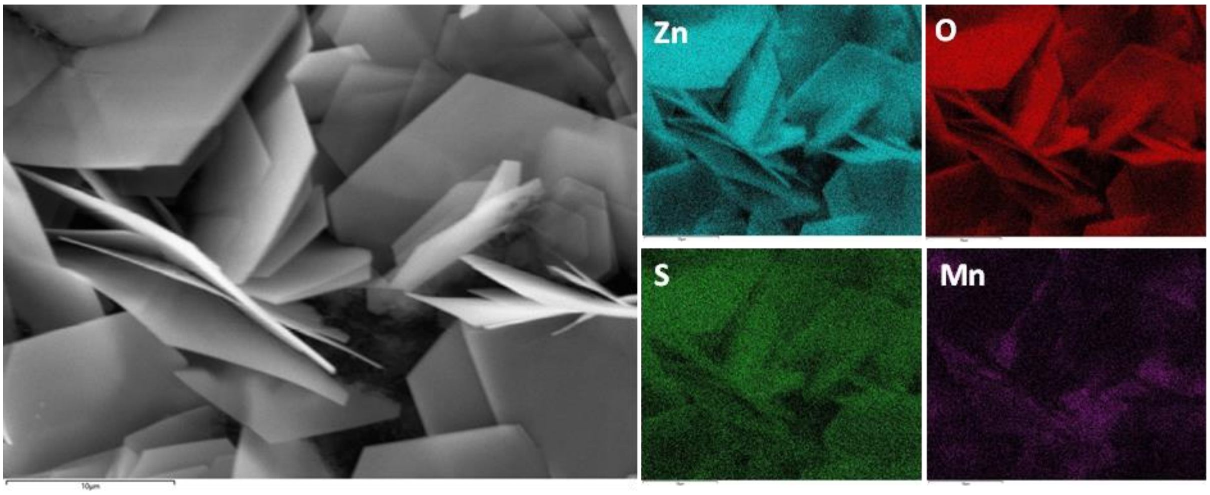

Figure S7. Energy-dispersive spectroscopy (EDS) analysis of the flake-like product on the electrode surface

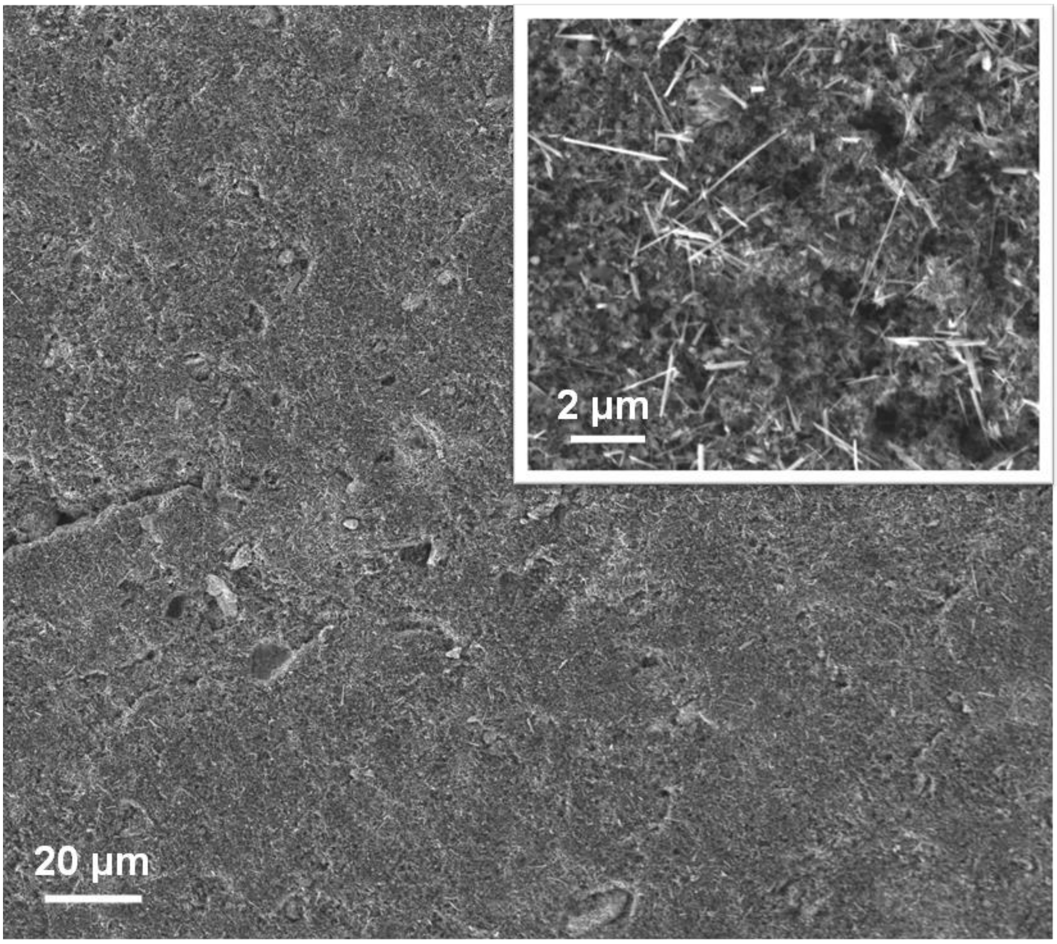

Figure S8. SEM images of electrode after the 1st discharge to 0.8 V and subsequent washing with acetic acid to remove the precipitate.

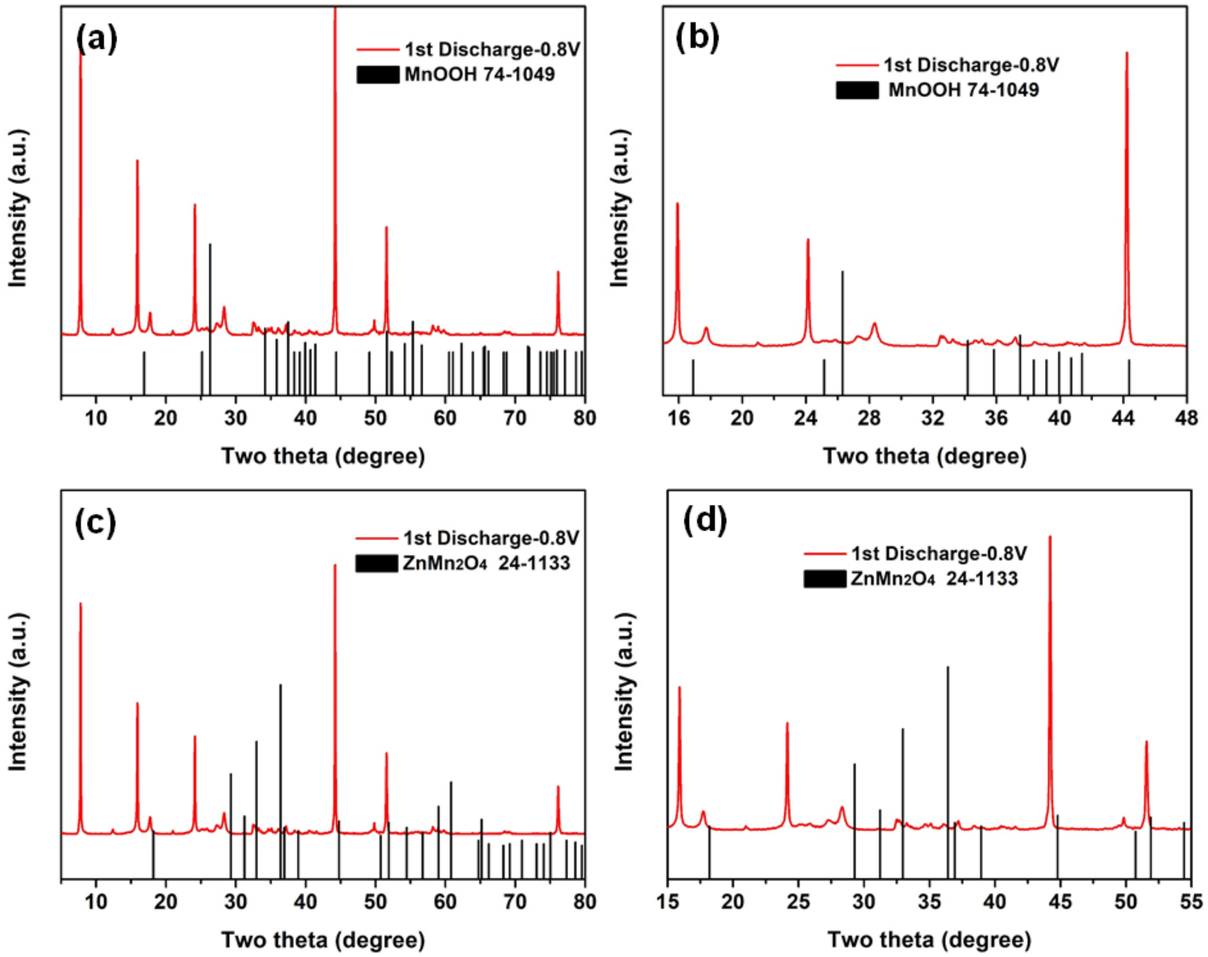

Figure S9. XRD pattern of discharged electrode compared with (a-b) MnOOH and (c-d) $ZnMn_2O_4$

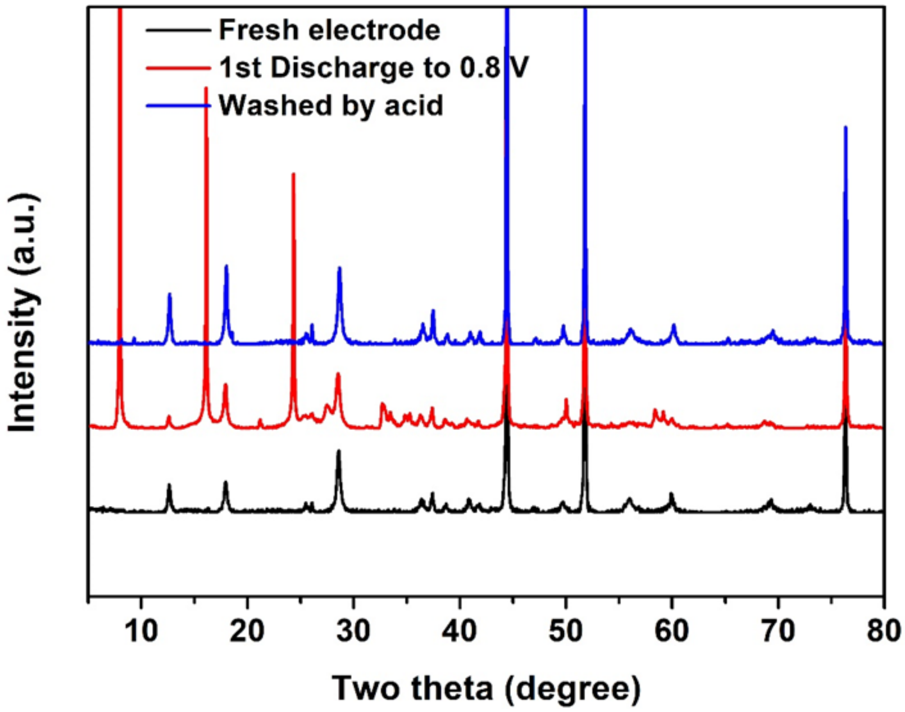

Figure S10. XRD patterns of fresh electrode and discharged electrode. The black line represents the fresh electrode. The red line is the electrode first discharged to 0.8 V.

After full discharge to 0.8 V, the discharged electrode was washed by weak acid to remove the precipitation on the electrode surface. The electrode washed by acid shows a similar XRD pattern to that of the fresh electrode. Surprisingly, only α-MnO_2 can be observed, and no evidence for Zn^{2+} or proton intercalation into α-MnO_2 can be found.

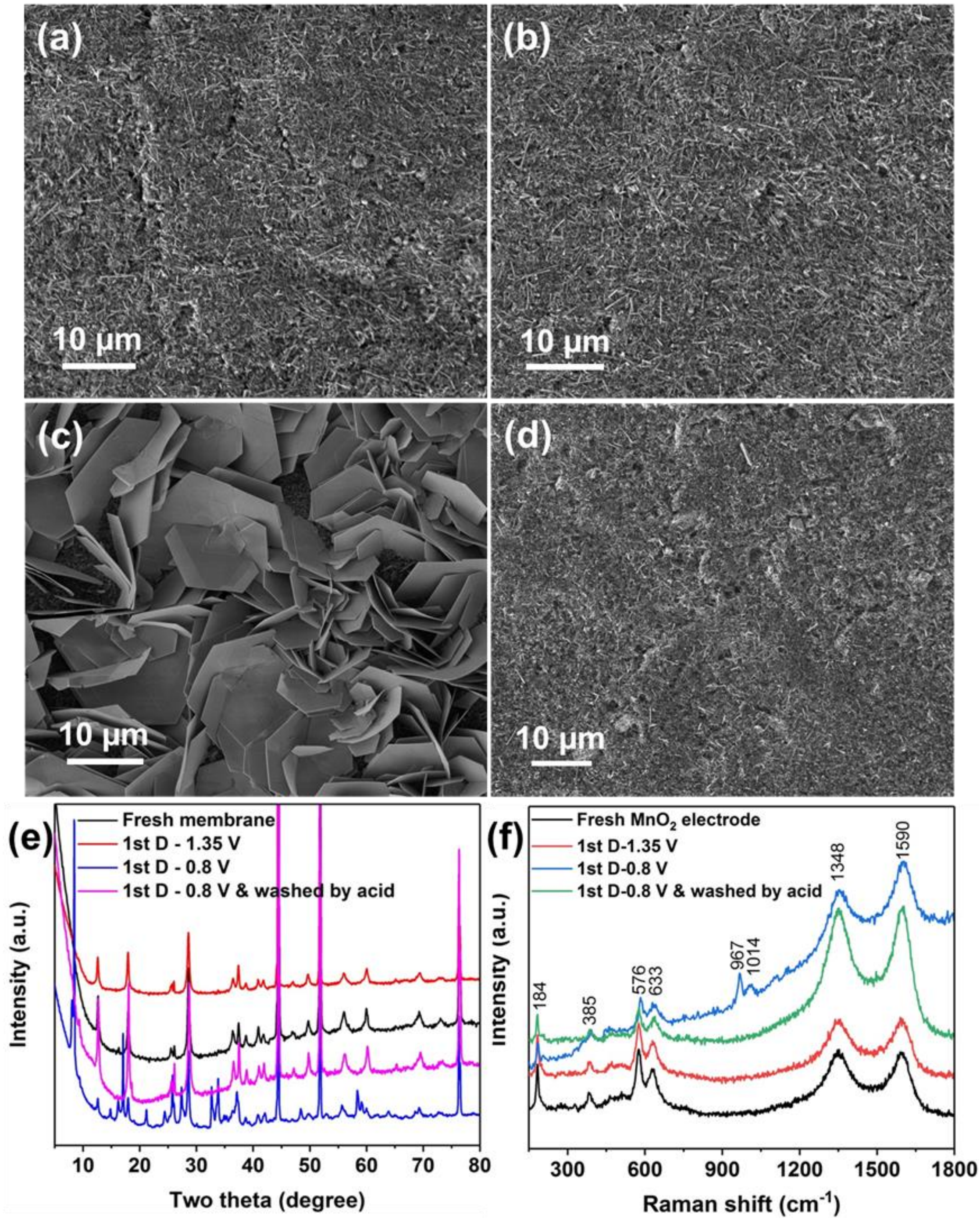

Figure S11. (a-b) SEM images of electrode in 2 M $ZnSO_4$ electrolyte: (a) original α-MnO_2, (b) 1st discharge to 1.35 V, (c) 1st discharge to 0.8 V, (d) 1st discharge to 0.8 V and washing with acetic acid to remove the precipitation, (e) XRD patterns of original α-MnO_2 electrode and discharged electrodes, (f) Raman spectra of the original α-MnO_2 electrode and discharged electrodes

The morphology of the MnO_2 electrode during the first discharge process in 2 M $ZnSO_4$ electrolyte was also observed by SEM. Figure S11a shows the morphology of the original MnO_2 electrode, exhibiting a clean surface. When the cell is discharged to 1.35 V, there is no obvious change on the electrode surface, shown in Figure S11b. But when the cell is further discharged to 0.8 V, some large flakes emerge on the electrode surface in Figure S11c. As shown in Figure S10d, the flakes on the electrode surface can be totally removed by acid. XRD analysis of the discharge product was conducted during the first discharge process. As shown in Figure S11e, when the cell is discharged to 1.35 V, there is no obvious variation compared with the XRD pattern of the fresh electrode. However, when the cell is further discharged to 0.8 V, the characteristic peaks of $Zn_4(OH)_6(SO_4)\cdot 5H_2O$ appear, which is consistent with the XRD results in 2 M

$ZnSO_4$ + 0.2 M $MnSO_4$. After the discharged electrode was treated by acid, the characteristic peaks belonging to $Zn_4(OH)_6(SO_4)\cdot5H_2O$ disappeared. Only α-MnO_2 can be observed, and no evidence for Zn^{2+} or proton intercalation into α-MnO_2 can be found. The discharge product was further investigated by Raman spectroscopy to reveal the structural evolution. As shown in Figure S11f, the original α-MnO_2 exhibits four main peaks at 184, 385, 576, and 633 cm^{-1}.[1, 8] The peaks at 633 cm^{-1} and 576 cm^{-1} arise from the symmetric Mn-O stretching vibrations in MnO_6 units. [5b, 8a] The translational motion of MnO_6 leads to an external vibration at 184 cm^{-1}. The peak located at 385 cm^{-1} is caused by the Mn-O bending vibrations.[8a] The peaks at 1348 cm^{-1} and 1590 cm^{-1} are assigned to the D band and G band of the conductive carbon added in the electrode. When the α-MnO_2 electrode is first discharged to 1.35 V, there is no significant change in the Raman spectrum. When it is further discharged to 0.8 V, two new peaks emerge at 967 cm^{-1} and 1014 cm^{-1}, which belongs to the vibrations of SO_4^{2-} in the zinc hydroxide sulfate hydrate.[9] After the discharged electrode is washed with acid, the peaks corresponding to SO_4^{2-} disappears and the whole spectrum is similar to that of the original MnO_2 electrode. No signals corresponding to MnOOH and $ZnMn_2O_4$ can be detected.

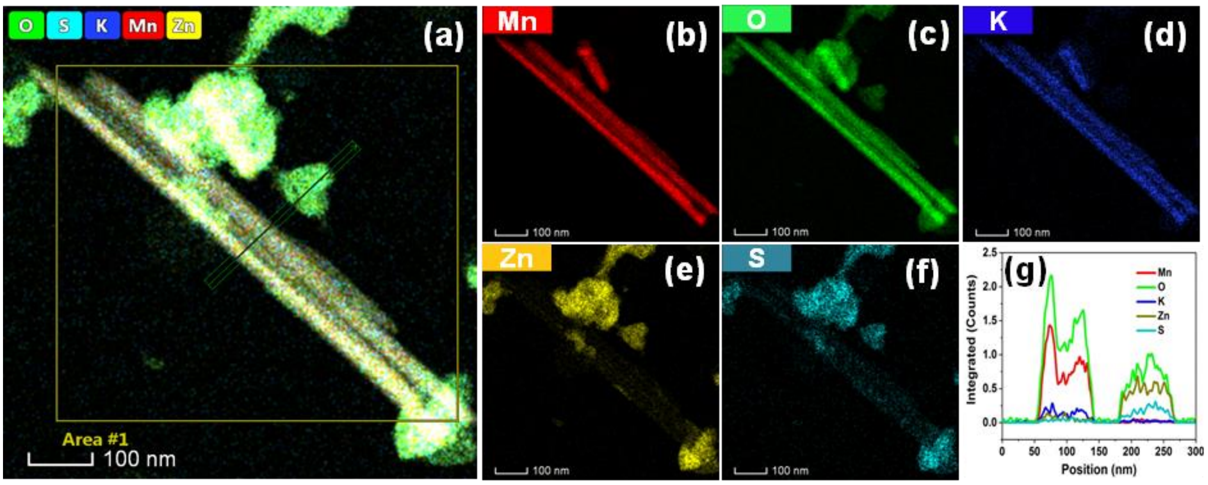

Figure S12. (a) STEM-HAADF image of MnO_2 electrode after 1st discharge, (b-f) STEM–EDS mappings of the elemental distributions of Mn, O, K, Zn and S, (g) line profiles of Mn, O, K, Zn and S extracted from the green rectangle in (a).

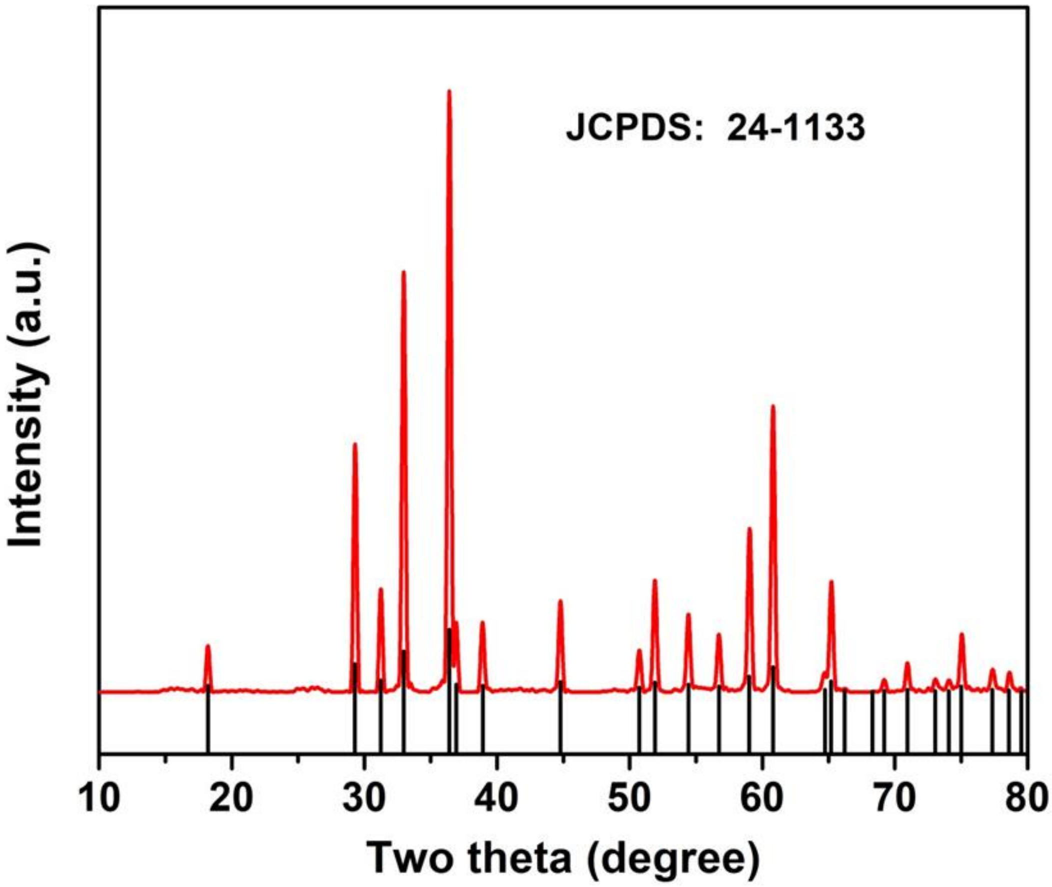

Figure S13. XRD pattern of the synthesized $ZnMn_2O_4$

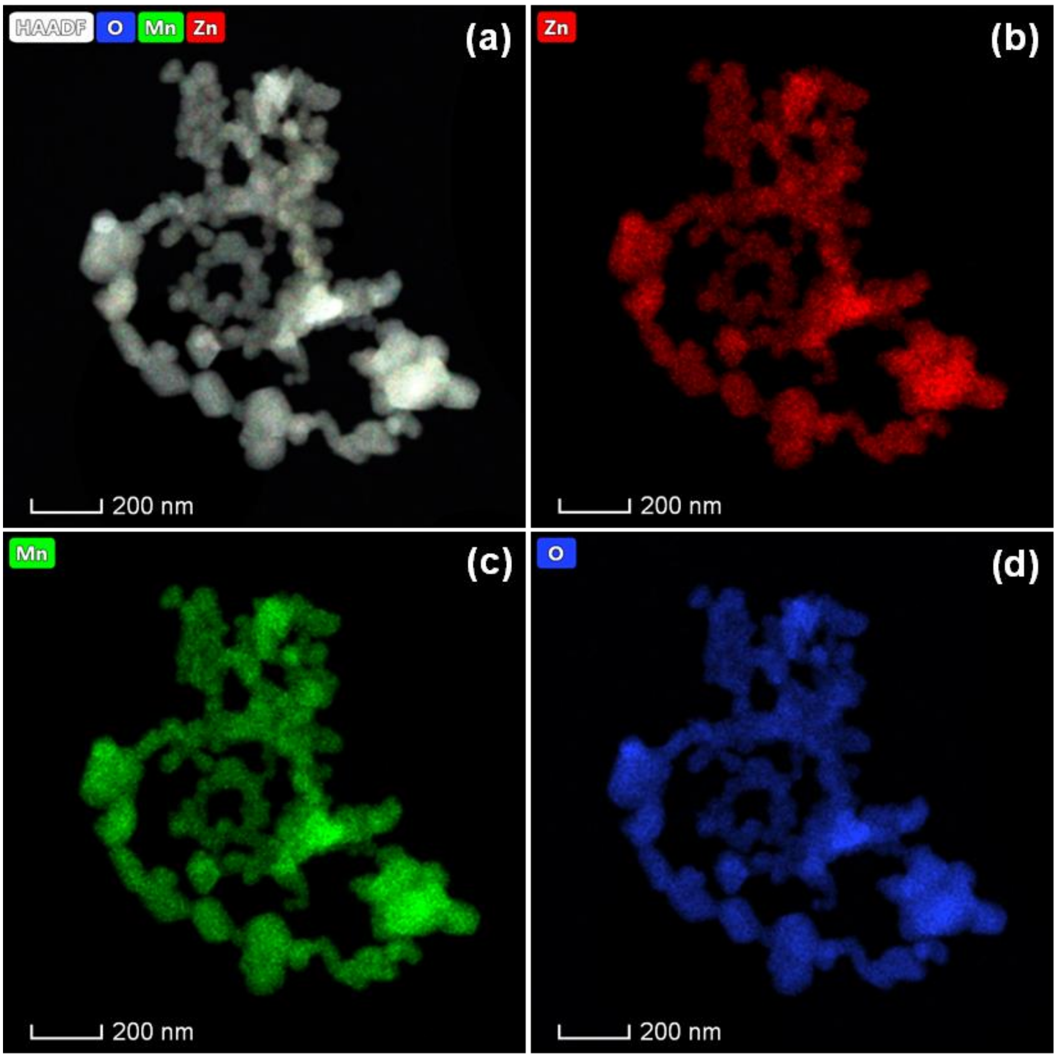

Figure S14. (a) STEM-HAADF image of $ZnMn_2O_4$ and STEM–EDS mappings of the elemental distributions of Mn, O and Zn in the $ZnMn_2O_4$

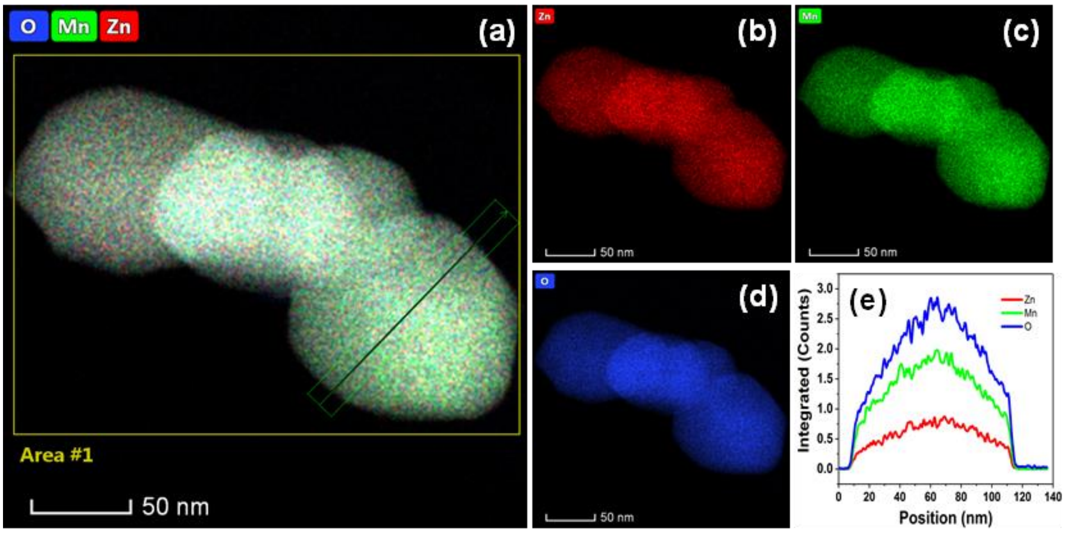

Figure S15. (a)STEM-HAADF image of $ZnMn_2O_4$, (b-d) STEM–EDS mappings of the elemental distributions of Zn, Mn and O, (e) line profiles of Zn, Mn and O extracted from green rectangle in (a)

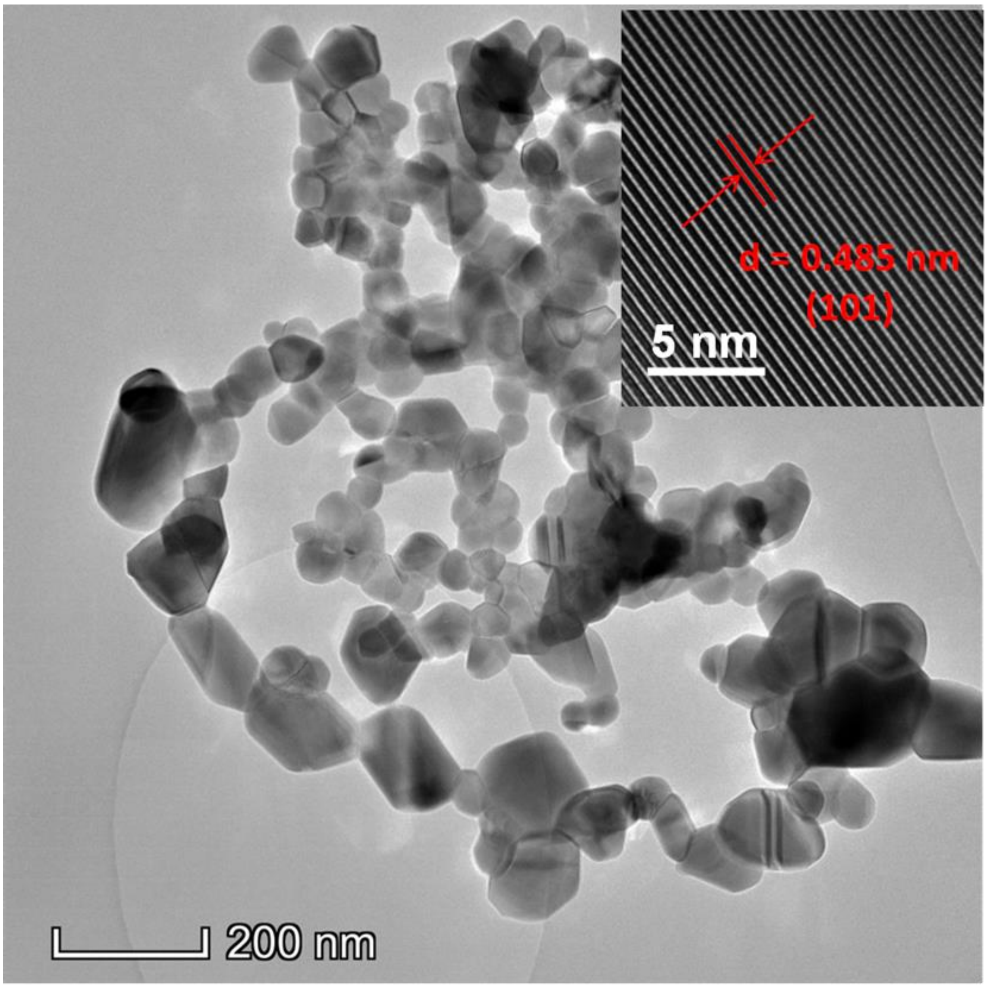

Figure S16. Representative TEM image of $ZnMn_2O_4$ (inset showing the HRTEM image)

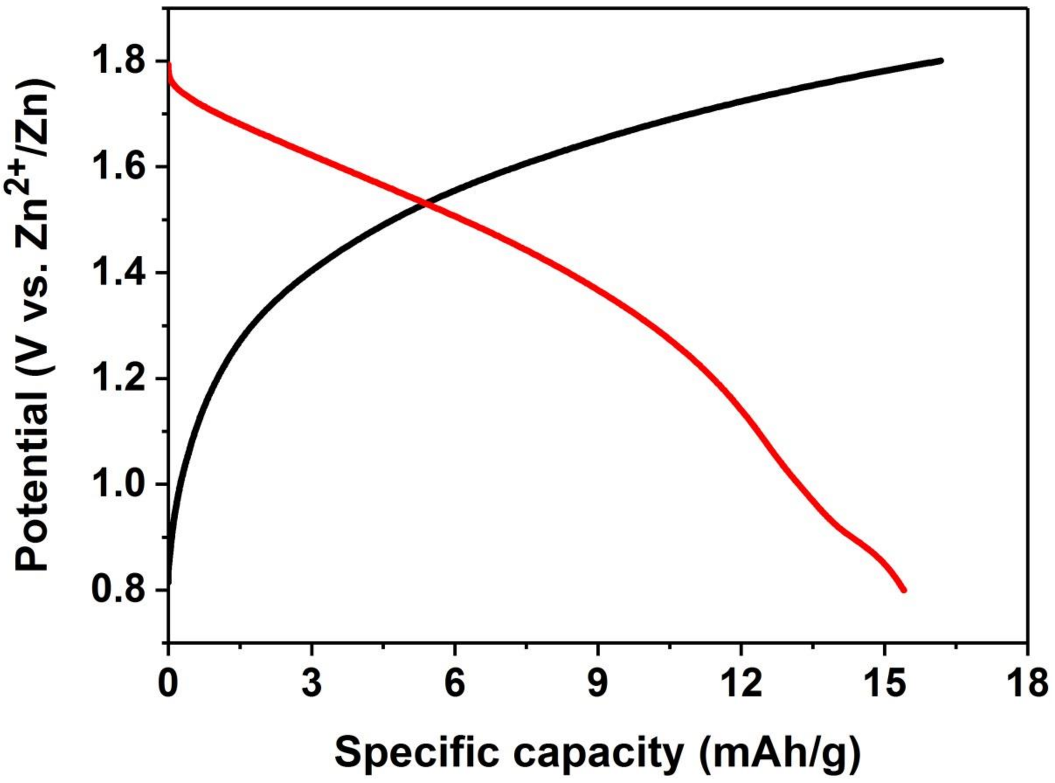

Figure S17. Typical galvanostatic charge-discharge curves of $ZnMn_2O_4$ electrode at a current density of 30 mA g^{-1}

Table S1. Comparison of different monovalent and multivalent metals[10]

Element	Standard potential (V vs. SHE)	Ionic radius (Å)	Hydrated ionic radius (Å)	Specific capacity (mAh/g)	Capacity density (mAh/cm^3)
Li	−3.040	0.76	3.40-3.82	3860	2061
Na	−2.713	1.02	2.76-3.60	1166	1129
K	−2.924	1.38	2.01-3.31	685	610
Mg	−2.356	0.72	3.00-4.70	2206	3834
Ca	−2.840	1.00	4.12-4.20	1337	2072
Zn	−0.763	0.75	4.04-4.30	820	5855
Al	−1.676	0.53	4.80	2980	8046

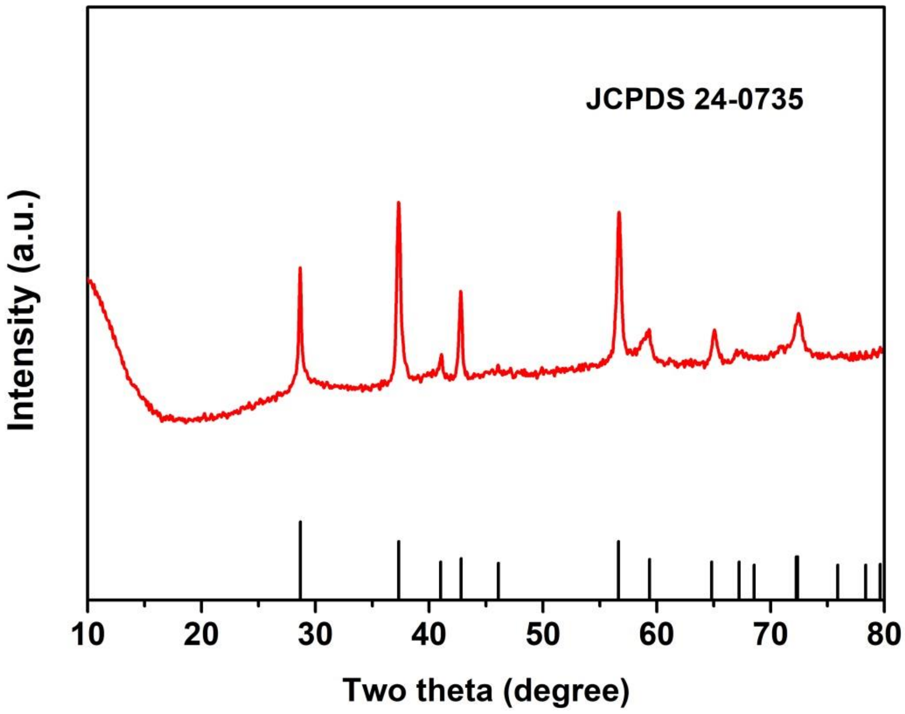

Figure S18. XRD Pattern of beta-MnO_2

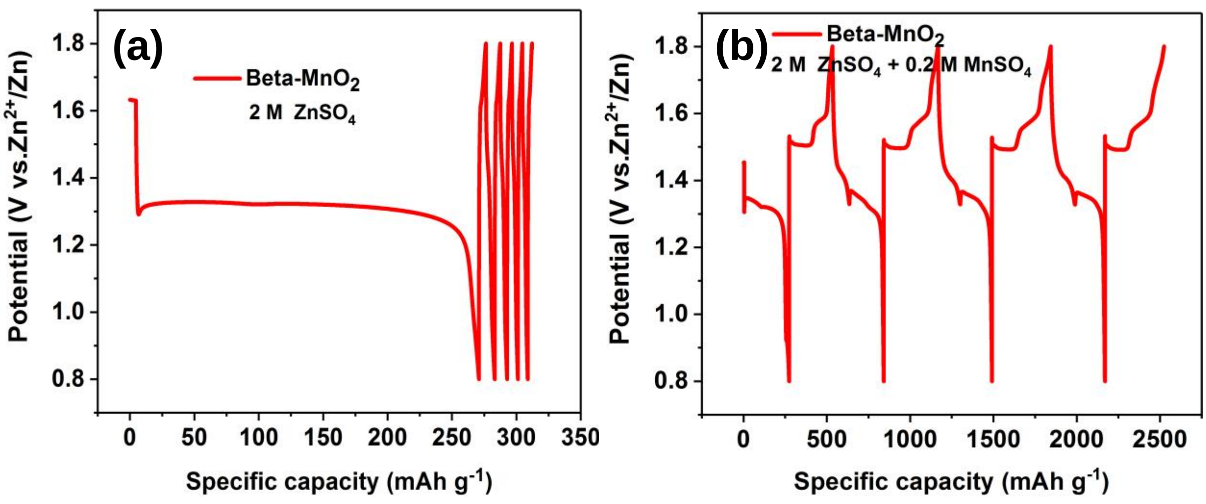

Figure S19. Typical galvanostatic charge-discharge curves of beta-MnO_2 (a) 2 M $ZnSO_4$ and (b) 2 M $ZnSO_4$ + 0.2 M $MnSO_4$

3. Potential conversion

$$MnO_2 + 4H^+ + 2e^- \rightleftharpoons Mn^{2+} + 2H_2O\ (E^\circ = 1.23\ \mathrm{V})$$

The concentration of Mn^{2+} is 0.2 M and the pH is measured to be about 5. So:

$$E = E^\circ - \frac{RT}{nF}\ln\frac{[Mn^{2+}]}{[H^+]^4} = 1.23 - \frac{8.314 \times 298.15}{2 \times 96485} \times \ln\frac{0.2}{[10^{-5}]^4} = 0.659\ \mathrm{V}$$

$$Zn^{2+} + 2e^- \rightleftharpoons Zn\ (E^\circ = -0.762\ \mathrm{V})$$

$$E = E^\circ - \frac{RT}{nF}\ln\frac{[Zn]}{[Zn^{2+}]} = -0.762 - \frac{8.314 \times 298.15}{2 \times 96485} \times \ln\frac{1}{2} = -0.754\ \mathrm{V}$$

So the required theoretical potential to electrodeposit MnO_2 is about 1.413 V (vs. Zn^{2+}/Zn) under our experimental conditions.

4. References for SI:

[1] S. Liang, F. Teng, G. Bulgan, R. Zong, Y. Zhu, *J. Phys. Chem.* **2008**, *112*, 5307-5315.

[2] X. Wang, Y. Li, *Chem. Eur. J.* **2003**, *9*, 300.

[3] R. Liu, Y. Yan, C. Ma, *Front Chem* **2018**, *6*, 69.

[4] W. Wei, X. Cui, W. Chen, D. G. Ivey, *Chem. Soc. Rev.* **2011**, *40*, 1697-1721.

[5] a) R. M. Freitas, T. A. G. Perilli, A. C. Q. Ladeira, *Journal of Chemistry* **2013**, *2013*, 1-8; b) S.-B. Ma, K.-Y. Ahn, E.-S. Lee, K.-H. Oh, K.-B. Kim, *Carbon* **2007**, *45*, 375-382.

[6] H. Pan, Y. Shao, P. Yan, Y. Cheng, K. S. Han, Z. Nie, C. Wang, J. Yang, X. Li, P. Bhattacharya, K. T. Mueller, J. Liu, *Nat. Energy* **2016**, *1*.

[7] T. Takashima, K. Hashimoto, R. Nakamura, *J. Am. Chem. Soc.* **2012**, *134*, 18153-18156.

[8] a) T. Gao, M. Glerup, F. Krumeich, R. Nesper, H. Fjellvåg, P. Norby, *J. Phys. Chem. C* **2008**, *112*, 13134-13140; b) T. Gao, H. Fjellvag, P. Norby, *Anal Chim Acta* **2009**, *648*, 235-239.

[9] a) Z. Du, J. Chen, W. Ye, J. Guo, X. Zhang, R. Zheng, *Sensors (Basel)* **2015**, *15*, 12377-12388; b) S. M. Angel, N. R. Gomer, S. K. Sharma, C. McKay, *Appl Spectrosc* **2012**, *66*, 137-150; c) K. Ben Mabrouk, T. H. Kauffmann, H. Aroui, M. D. Fontana, *J. Raman Spectrosc* **2013**, *44*, 1603-1608.

[10] M. Song, H. Tan, D. Chao, H. J. Fan, *Adv. Funct. Mater.* **2018**, *28*, 1802564.
